# Intermittent fasting uncovers and rescues cognitive phenotypes in PTEN neuronal haploinsufficient mice

**DOI:** 10.1038/s41598-018-26814-6

**Published:** 2018-06-05

**Authors:** J. V. Cabral-Costa, D. Z. Andreotti, N. P. Mello, C. Scavone, S. Camandola, E. M. Kawamoto

**Affiliations:** 1 Laboratory of Molecular and Functional Neurobiology, Department of Pharmacology, Institute of Biomedical Sciences, University of São Paulo, São Paulo, Brazil; 2 Laboratory of Molecular Neuropharmacology, Department of Pharmacology, Institute of Biomedical Sciences, University of São Paulo, São Paulo, Brazil; 3 Laboratory of Neurosciences, NIA, NIH, Baltimore, MD USA

## Abstract

Phosphatase and tensin homolog (PTEN) is an important protein with key modulatory functions in cell growth and survival. PTEN is crucial during embryogenesis and plays a key role in the central nervous system (CNS), where it directly modulates neuronal development and synaptic plasticity. Loss of PTEN signaling function is associated with cognitive deficits and synaptic plasticity impairment. Accordingly, *Pten* mutations have a strong link with autism spectrum disorder. In this study, neuronal *Pten* haploinsufficient male mice were subjected to a long-term environmental intervention – intermittent fasting (IF) – and then evaluated for alterations in exploratory, anxiety and learning and memory behaviors. Although no significant effects on spatial memory were observed, mutant mice showed impaired contextual fear memory in the passive avoidance test – an outcome that was effectively rescued by IF. In this study, we demonstrated that IF modulation, in addition to its rescue of the memory deficit, was also required to uncover behavioral phenotypes otherwise hidden in this neuronal *Pten* haploinsufficiency model.

## Introduction

Phosphatase and tensin homolog (PTEN) was originally characterized as a tumor suppressor^1,2^ as it is commonly deleted in several human tumors^3–5^. The most well described function of PTEN is as a lipid and protein phosphatase that classically converts 3,4,5-phosphatidylinositol trisphosphate into 4,5-phosphatidylinositol bisphosphate, thus acting as a negative modulator of AKT signaling activation^6^. PTEN plays an important modulatory role in cell growth, proliferation and survival and is critical to key processes in animal development^7^. Indeed, PTEN knockout mice die precociously during embryogenesis^8–11^. Recently, PTEN was shown to potentially act through phosphatase-independent mechanisms^12,13^, thus expanding its influence beyond the AKT signaling pathway.

In the central nervous system (CNS), PTEN is widely expressed in neurons, particularly in dendritic spines, across several brain areas, including the cerebellum, cortex, hippocampi and olfactory bulb^14^. PTEN is crucial for mature neuron survival, playing an important role in neurite extension^15^ and modulating cell number, size and migration properties^16–19^. *Pten* deletion induces an increase in the size and number of dendrite ramifications and synapses^20–22^, culminating in functional synaptic plasticity impairment^23,24^. *Pten* mutations have been linked to cognitive deficits in humans^25^. Accordingly, mice with neuronal *Pten* deletion showed impaired social interaction and increased anxiety behavior^20,26^, thus validating their use to elucidate the role of PTEN in the CNS and cognition, particularly in the study of autism spectrum disorders.

Intermittent fasting (IF) is an environmental intervention with known modulatory and neuroprotective effects^27^. The regimen consists of alternating days of free access to food with those of complete fasting long term, thus not necessarily affecting the total amount of food consumed but rather the intake frequency^28^. In the CNS, IF was shown to improve learning and memory from rats and mouse models through humans^29^ and to modulate cognition and synaptic plasticity through changes in the expression profile of glutamatergic ionotropic receptors in mice^30^. Therefore, IF is a potent environmental intervention that can be used as a tool to stimulate the CNS as well as potentially rescue impaired function.

In this study, we examined the behavioral profile of male mice with neuronal *Pten* haploinsufficiency under IF conditions. We also assessed markers of AKT signaling activity and synaptic and glutamatergic receptor profiles.

## Results

### Intermittent fasting induced an intermittent loss of body mass and a decrease in total food consumption

**Figure 1:**
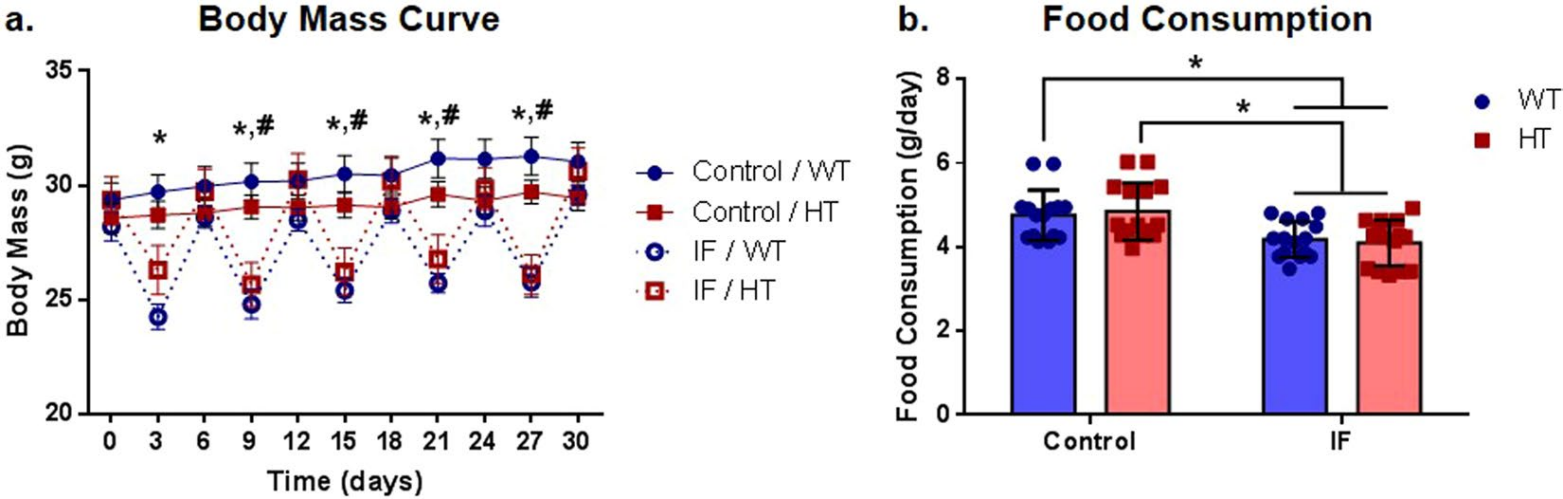
Body mass and food consumption during the intermittent fasting protocol. (**a**) Body mass curve of the first 30 days of intermittent fasting, two-way ANOVA with repeated measures followed by Holm-Sidak’s post hoc test, ^*^P ≤ 0.0001 for C/WT x IF/WT, ^#^P ≤ 0.05 for C/HT x IF/HT, n = 14 (IF/HT), 15 (C/WT, C/HT), and 16 (IF/WT); (**b**) average food consumption, two-way ANOVA followed by Holm-Sidak’s post hoc test, ^*^P ≤ 0.05, n = 15 (C/WT, IF/WT, IF/HT) and 16 (C/HT).

When assessed after a period of fasting, animals under IF showed a significantly lower body mass than control animals (P ≤ 0.0001 for control (C)/wild-type (WT) × IF/WT; P ≤ 0.05 for C/heterozygous (HT) × IF/HT), an intermittent effect that was rescued at each following period of *ad libitum* food offering (Fig. 1a). Interestingly, there was no significant effect on body mass variation induced by the IF regimen (F (1, 71) = 0.3694, P = 0.5453 for the treatment factor; F (1, 71) = 2.289, P = 0.1347 for the genotype factor) (Supplementary Fig. S1).

Regardless of the genotype, animals under IF consumed significantly less food than control animals (F (1, 56) = 20.45, P ≤ 0.0001 for the treatment factor) (Fig. 1b). However, this reduction in food intake (on average, 12.1% for C/WT × IF/WT and 15.5% for C/HT × IF/HT) was remarkably modest, considering that the food intake of IF groups was restricted to fed days.

### HT animals displayed a significant increase in total brain and cortical mass

**Figure 2:**
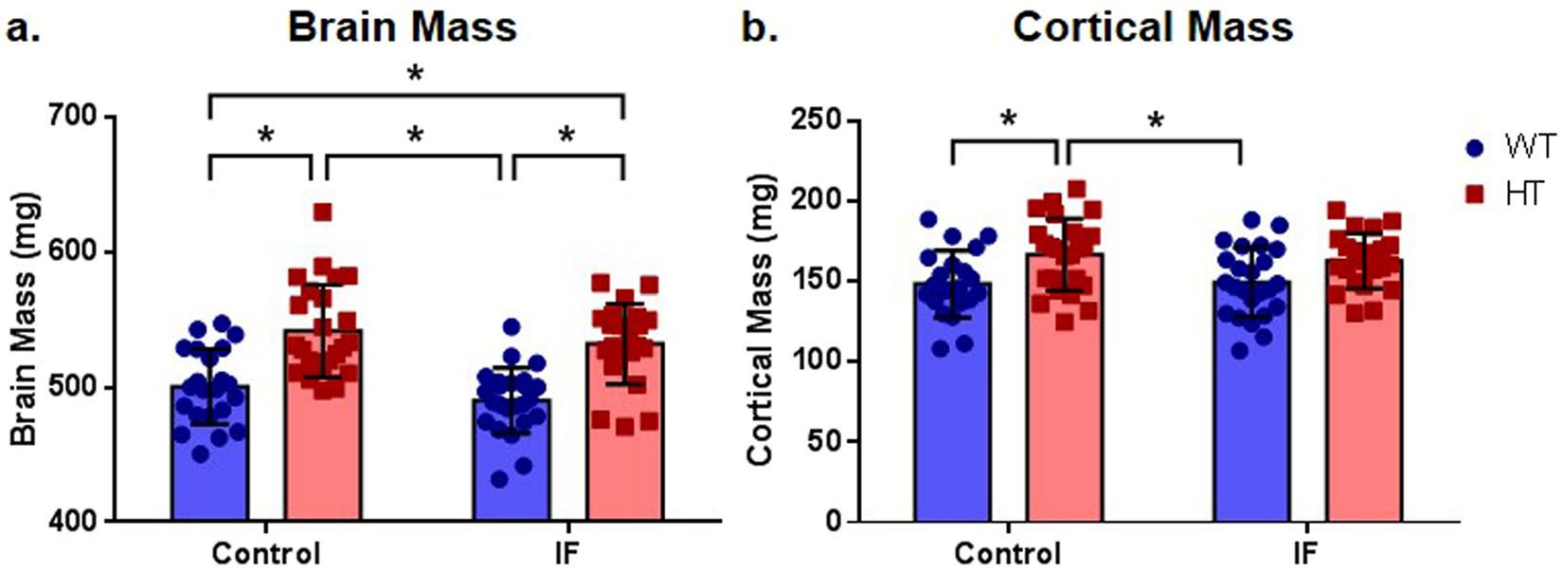
Neuronal PTEN deletion induced macrocephaly. (**a**) Total brain mass, two-way ANOVA followed by Holm-Sidak’s post hoc test, ^*^P ≤ 0.01, n = 22 (C/WT, IF/HT) and 24 (C/HT, IF/WT); (**b**) cortical mass, two-way ANOVA followed by Holm-Sidak’s post hoc test, ^*^P  ≤ 0.05, n = 21 (C/WT), 22 (IF/HT) and 24 (C/HT, IF/WT).

*Pten* haploinsufficiency caused an increase in total brain mass compared to the WT condition (F (1, 88) = 46.42, P ≤ 0.0001 for the genotype factor), although no differences were observed when upon comparison of the control and IF groups (F (1,88) = 2.626, P = 0.1087 for the treatment factor) (Fig. 2a). This difference was not due to a greater total body mass (F (1, 88) = 0.4222, P = 0.5175 for the treatment factor; F (1, 88) = 0.1252, P = 0.7243 for the genotype factor) (Supplementary Fig. S2).

The changes in brain mass resulted from an increase in cortical mass (F (1, 87) = 13.06, P = 0.0005 for the genotype factor), with control HT animals showing significantly higher values than WT mice (control and IF) (P = 0.0246 for C/WT × C/HT and for C/HT × IF/WT) (Fig. 2b). However, neither the cerebellum (F (1, 86) = 1.052, P = 0.3079 for the treatment factor; F (1, 86) = 0.5044, P = 0.4795 for the genotype factor) (Supplementary Fig. S2) nor the hippocampus (F (1, 82) = 2.437, P = 0.1224 for the treatment factor; F (1, 82) = 0.5252, P = 0.4707 for the genotype factor) (Supplementary Fig. S2) showed a difference in mass.

### Intermittent fasting increased the open arm exploration frequency in the elevated plus maze in HT animals

**Figure 3:**
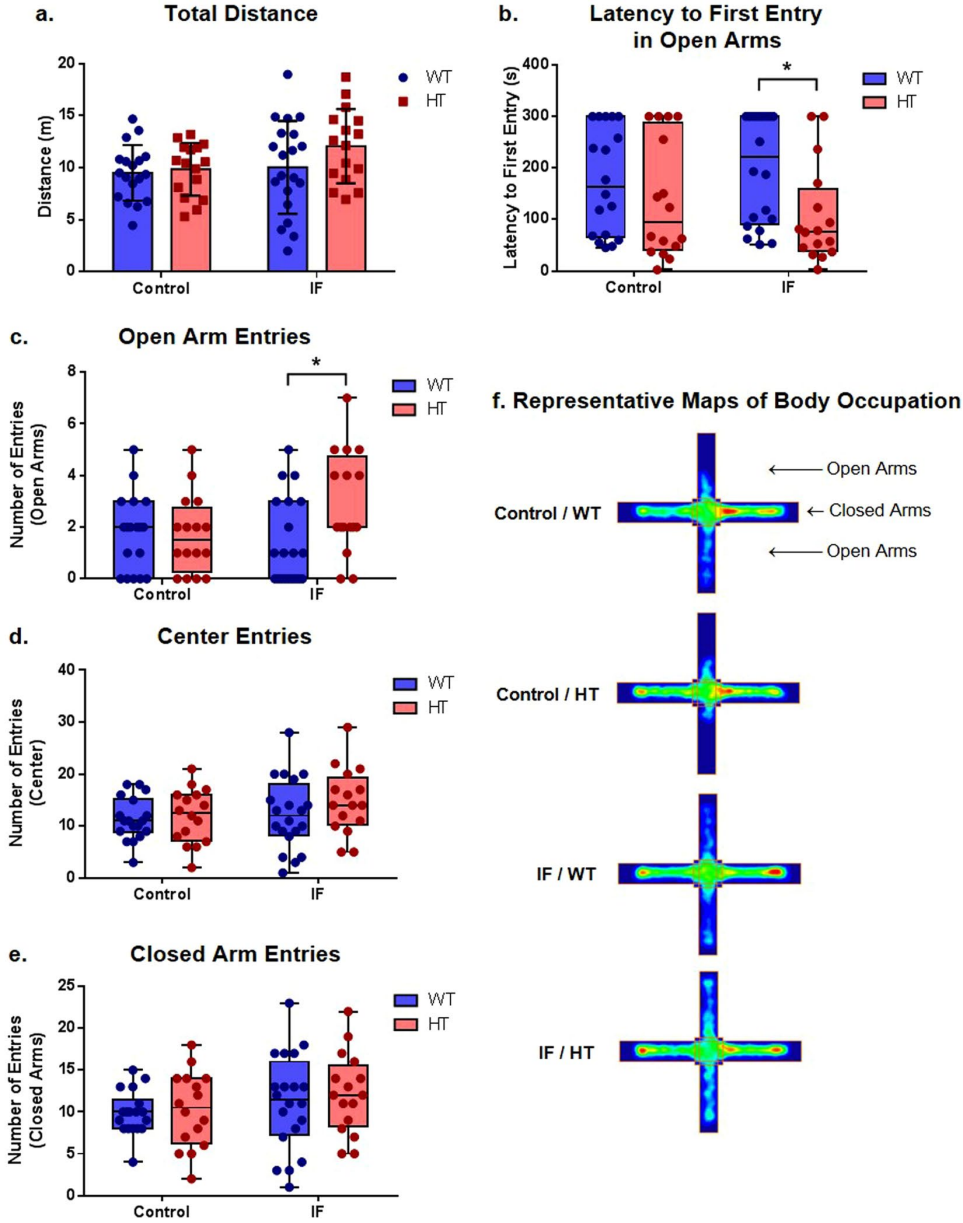
Intermittent fasting increased the exploration of open arms in HT animals in the elevated plus maze assay. (**a**) Total distance travelled, two-way ANOVA followed by Holm-Sidak’s post hoc test, P > 0.05, n = 16 (C/HT, IF/HT), 18 (C/WT), and 20 (IF/WT); (**b**) latency to first entry in open arms, Kruskal-Wallis test, ^*^P ≤ 0.05, n = 16 (C/HT, IF/HT), 18 (C/WT), and 20 (IF/WT); (**c**) number of entries in open arms, Kruskal-Wallis test, ^*^P ≤ 0.05, n = 16 (C/HT, IF/HT), 18 (C/WT), and 20 (IF/WT); (**d**) number of entries in central area, Kruskal-Wallis test, P > 0.05, n = 16 (C/HT, IF/HT), 18 (C/WT), and 20 (IF/WT); (**e**) number of entries in closed arms, Kruskal-Wallis test, P > 0.05, n = 16 (C/HT, IF/HT), 18 (C/WT), and 20 (IF/WT); (**f**) representative heat map plot for animal movement in the apparatus.

Analysis of the elevated plus maze test showed no significant differences between groups in locomotion as determined by the total distance travelled in the apparatus (F (1, 66) = 2.726, P = 0.1035 for the treatment factor; F (1, 66) = 2.052, P = 0.1567 for the genotype factor) (Fig. 3a). Interestingly, intermittent fasting in HT animals decreased the latency to first entry (P = 0.0279 for IF/WT × IF/HT) (Fig. 3b, Supplementary Fig. S3) and increased the number of entries (P = 0.0453 for IF/WT × IF/HT) (Fig. 3c, Supplementary Fig. S3) into the open arms. No differences were found in the frequency of exploration of the central area (Fig. 3d, Supplementary Fig. S3) or closed arms (Fig. 3e, Supplementary Fig. S3). Representative exploration profiles for the various groups are shown in the heat map plot in Fig. 3f.

### Intermittent fasting did not alter locomotion but exerted a decrease in time in central area exploration in HT animals

**Figure 4:**
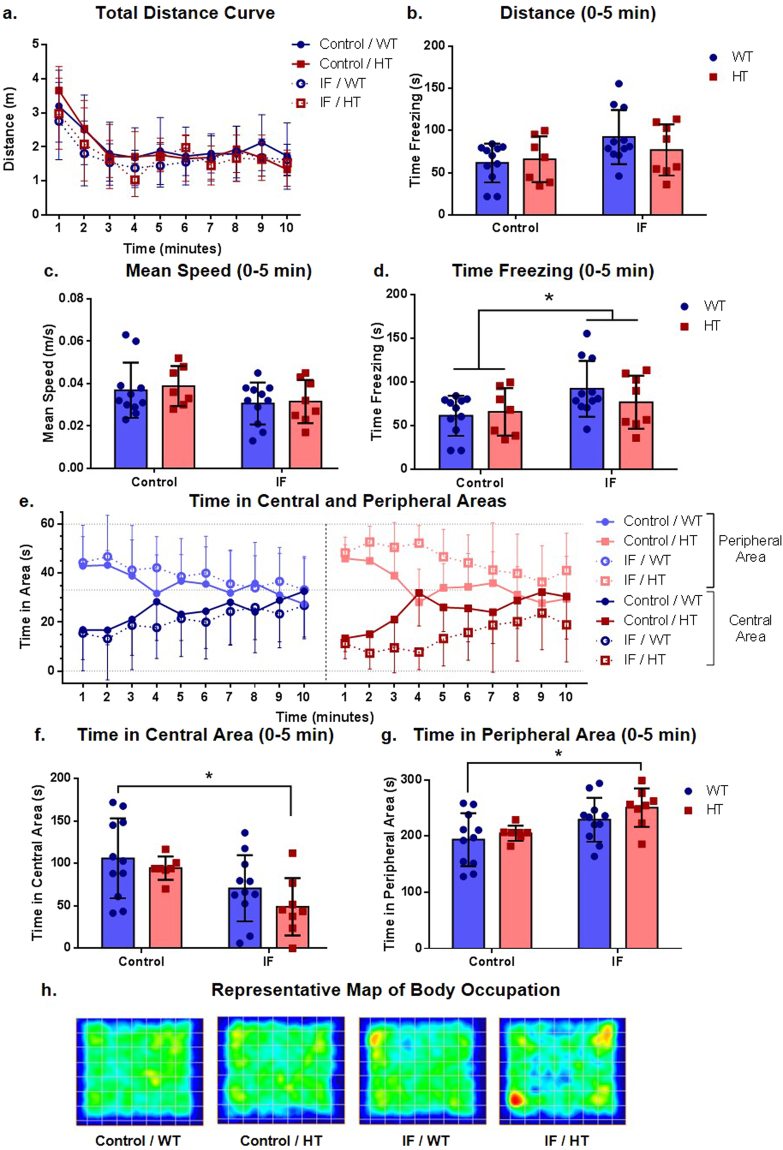
Intermittent fasting induced a decrease in central area exploration in HT animals in the open field test. (**a**) Curve of distance travelled, two-way ANOVA followed by Holm-Sidak’s post hoc test, P > 0.05, n = 8 (C/HT, IF/HT), 11 (C/WT), and 12 (IF/WT); (**b**) distance travelled in first 5 min, two-way ANOVA followed by Holm-Sidak’s post hoc test, P > 0.05, n = 7 (C/HT), 8 (IF/HT) and 11 (C/WT, IF/WT); (**c**) mean speed in first 5 min, two-way ANOVA followed by Holm-Sidak’s post hoc test, P > 0.05, n = 7 (C/HT), 8 (IF/HT) and 11 (C/WT, IF/WT); (**d**) total time of freezing behavior in first 5 min, two-way ANOVA followed by Holm-Sidak’s post hoc test, P ≤ 0.05 for the treatment factor, n = 7 (C/HT), 8 (IF/HT) and 11 (C/WT, IF/WT); (**e**) curves for time in central and peripheral areas; (**f**) time in central area in first 5 min, two-way ANOVA followed by Holm-Sidak’s post hoc test, ^*^P ≤ 0.05, n = 7 (C/HT), 8 (IF/HT) and 11 (C/WT, IF/WT); (**g**) time in peripheral area in first 5 min, two-way ANOVA followed by Holm-Sidak’s post hoc test, ^*^P ≤ 0.05, n = 7 (C/HT), 8 (IF/HT) and 11 (C/WT, IF/WT); (**h**) representative heat map plot for animal movement in the apparatus.

Animals from all groups showed a decline in locomotor activity in up to 5 min (Fig. 4a). Because they reached a stable state after 5 min exploring the apparatus, we limited the analysis of the different behavioral parameters to this period of time. All groups showed analogous exploratory activity (F (3, 35) = 0.9707, P = 0.4175 for the group factor) (Fig. 4a) and statistically similar travelled distances (F (1,33) = 3.575, P = 0.0675 for the treatment factor; F (1, 33) = 0.1699, P = 0.6829 for the genotype factor) (Fig. 4b) and mean speeds (F (1,33) = 3.460, P = 0.0718 for the treatment factor; F (1, 33) = 0.1472, P = 0.7037 for the genotype factor) (Fig. 4c). Freezing response in IF groups was significantly greater than in control groups (F (1, 33) = 4.886, P = 0.0341 for the treatment factor) (Fig. 4d).

Regarding the exploration patterns in the open field, animals tended to primarily explore the peripheral area, with an increase in the time spent in the central area over time (Fig. 4e). Notably, a delay in entering the central area was seen for IF HT animals (Fig. 4e). Indeed, the IF HT group spent significantly less time in the central area than the control WT group (F (1, 33) = 10.33, P = 0.00029 for the treatment factor; P = 0.0149 for C/WT × IF/HT) (Fig. 4f) and consequently more time in the periphery (F (1, 33) = 10.33, P = 0.00029 for the treatment factor; P = 0.0154 for C/WT × IF/HT) (Fig. 4g). Representative exploratory patterns for the different groups are presented in the heat map plot in Fig. 4h.

### Intermittent fasting did not modify spatial learning and memory

**Figure 5:**
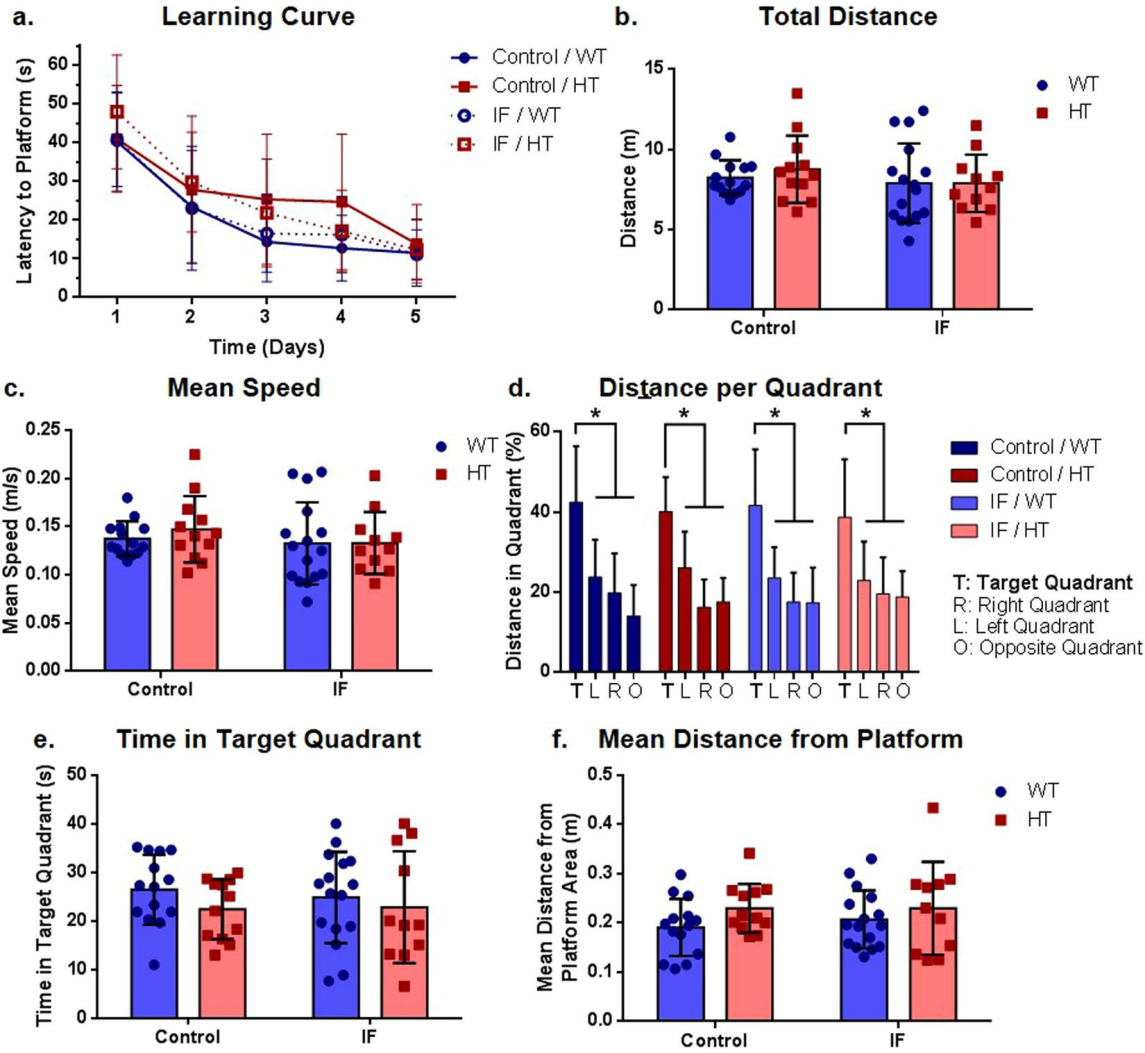
Neither treatment nor genotype significantly altered short-term spatial reference memory in the Morris water maze assay. (**a**) Learning curve of platform location, two-way ANOVA with repeated measures followed by Holm-Sidak’s post hoc test, P > 0.05, n = 12 (C/HT, IF/HT), 15 (C/WT) and 16 (IF/WT); (**b**) total distance travelled, two-way ANOVA followed by Holm-Sidak’s post hoc test, P > 0.05, n = 11 (IF/HT), 12 (C/HT), 14 (C/WT) and 16 (IF/WT); (**c**) mean swimming speed, two-way ANOVA followed by Holm-Sidak’s post hoc test, P > 0.05, n = 11 (IF/HT), 12 (C/HT), 14 (C/WT) and 16 (IF/WT); (**d**) distance travelled in each quadrant of the Morris water maze, one-way ANOVA, ^*^P ≤ 0.001, n = 12 (C/HT, IF/HT), 15 (C/WT) and 16 (IF/WT); (**e**) time spent in target quadrant, two-way ANOVA followed by Holm-Sidak’s post hoc test, P > 0.05, n = 11 (IF/HT), 12 (C/HT), 14 (C/WT) and 16 (IF/WT); (**f**) mean distance from platform area, two-way ANOVA followed by Holm-Sidak’s post hoc test, P > 0.05, n = 11 (IF/HT), 12 (C/HT), 14 (C/WT) and 16 (IF/WT).

Spatial memory was assessed using the Morris water maze assay. All animals showed a significant decrease in the latency to platform over time (F (4, 204) = 75.95, P ≤ 0.0001 for the time factor), but there were no significant differences in learning between groups (F (3, 51) = 1.548, P = 0.2135 for the group factor) (Fig. 5a).

Short-term memory was tested by probing the animals in the water maze in the absence of the platform 4 h after the last learning trial. There were no significant differences between the total distance travelled (F (1, 49) = 1.234, P = 0.2721 for the treatment factor; F (1, 49) = 0.2385, P = 0.6275 for the genotype factor) (Fig. 5b) or mean swimming speed (F (1, 49) = 1.043, P = 0.3122 for the treatment factor; F (1, 49) = 0.2912, P = 0.5919 for the genotype factor) (Fig. 5c). All groups showed a significant preference for the target quadrant (F (3, 153) = 51.14, P ≤ 0.0001 for the quadrant factor; P < 0.01 for all comparisons between the target quadrant and the right, left or opposite quadrants respective to each group) (Fig. 5d), indicating a similar consolidation and evocation of short-term spatial memory. Indeed, there was no difference between groups in any parameter analyzed, including time in target quadrant (F (1, 49) = 0.06066, P = 0.8065 for the treatment factor; F (1, 49) = 1.587, P = 0.2138 for the genotype factor) (Fig. 5e), mean distance from platform (F (1, 49) = 0.2060, P = 0.6519 for the treatment factor; F (1, 49) = 2.867, P = 0.0968 for the genotype factor) (Fig. 5f), number of entries into the target quadrant (P > 0.05 for all comparisons) (Supplementary Fig. S4), or number of entries into the platform area (P > 0.05 for all comparisons) (Supplementary Fig. S4).

Comparable profiles were observed among all groups (F (3, 30) = 2.345, P = 0.0927 for the group factor) and between probes (F (4, 120) = 1.052, P = 3835 for the time factor) in the memory extinction test (Supplementary Fig. S4). With regard to spatial working memory, animals showed a decrease in the latency to the platform over consecutive trials (F (3, 87) = 21.07, P ≤ 0.0001 for the time factor), although no differences between groups were observed (F (3, 29) = 1.730, P = 0.1828 for the group factor) (Supplementary Fig. S4). Similar results were obtained in the novel object recognition assay (P > 0.05 for all comparisons) (Supplementary Fig. S5).

### Intermittent fasting rescued the fear-associated memory deficit displayed by HT animals in the passive avoidance test

**Figure 6:**
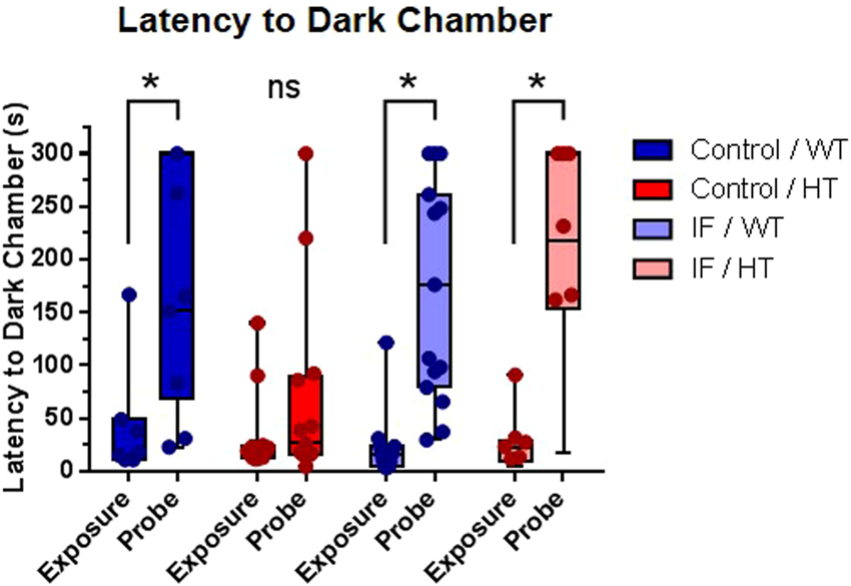
Intermittent fasting rescued the fear memory deficit of HT animals in the passive avoidance test. Latency to dark chamber in the exposure and probe stages, Kruskal-Wallis test, ^*^P ≤ 0.01, n = 10 (IF/HT), 11 (C/WT), 13 (C/HT) and 15 (IF/WT).

Fear-associated memory was assessed using the passive avoidance test. All groups showed a similar baseline latency to move to the dark chamber in the exposure stage (Fig. 6, Supplementary Fig. S6). WT animals showed a significant increase in latency in the probe stage regardless of the diet regimen [control (P = 0.0057 for C/WT Exposure × Probe); IF (P ≤ 0.0001 for IF/WT Exposure × Probe)]. On the other hand, no significant change in the latency to the dark chamber was found in control HT animals (P > 0.9999 for C/HT Exposure × Probe), an effect that was rescued by IF (P = 0.0013 for IF/HT Exposure × Probe).

### Expression of PTEN but not all major downstream markers was altered in HT animals

**Figure 7:**
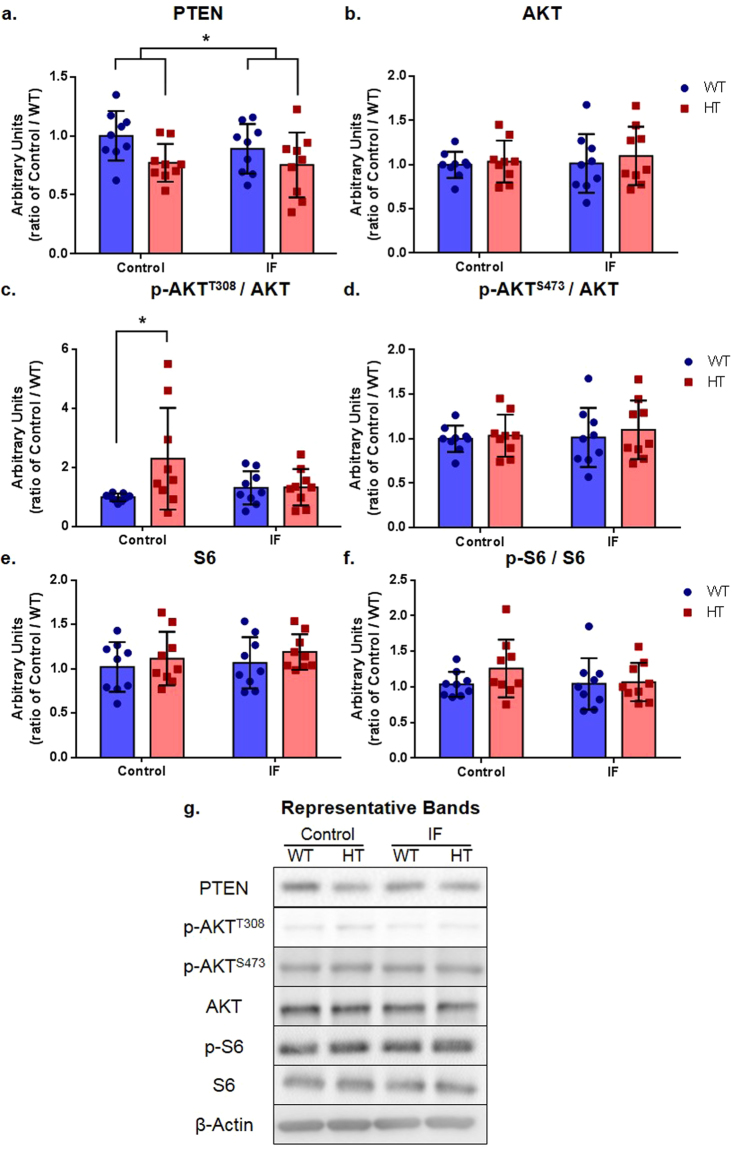
PTEN/AKT signaling pathway. Protein levels assessed through western blotting. (**a**) PTEN; (**b**) total AKT; (**c**) p-AKT^T308^ normalized by total AKT; (**d**) p-AKT^S473^ normalized by total AKT; (**e**) total S6; (**f**) p-S6 normalized by total S6; (**g**) representative bands. Two-way ANOVA followed by Holm-Sidak’s post hoc test, ^*^F (1,32) = 6.356, P = 0.0169 for the genotype factor in the two-way ANOVA test in (**a**). ^*^P  ≤ 0.05 for C/WT × C/HT in (**c**), n = 9 in all groups. Full length blots are provided in the Supplementary Figs S8 and S10.

Results from statistical analyses of western blotting data are grouped on Table 1. HT animals showed significantly lower PTEN levels in the brain cortex than WT animals (Fig. 7a, Supplementary Fig. S7), with no IF-associated changes. AKT expression was similar across groups (Fig. 7b, Supplementary Fig. S7), but control HT animals showed significantly higher levels of p-AKT^T308^ than control WT mice (Fig. 7b, Supplementary Fig. S7), an effect that was absent in the IF HT group. These data indicate that a PI3K-dependent overactivation of AKT, induced by neuronal *Pten* haploinsufficiency, might be rescued by IF. Notably, no differences were observed in phosphorylation at the mTOR-dependent AKT phosphorylation site (p-AKT^S473^) (Fig. 7d, Supplementary Fig. S7) nor in the total expression of its downstream target S6 (Fig. 7e, Supplementary Fig. S7) or its phosphorylated form, p-S6 (Fig. 7f, Supplementary Fig. S7)). Levels of the glutamatergic ionotropic receptors AMPA (Supplementary Figs S8 and S9), NR1 (Supplementary Figs S8 and S9), NR2a (Supplementary Figs S8 and S9), and NR2b (Supplementary Figs S8 and S9)) and the synaptic markers PSD-95 (Supplementary Figs S8 and S9) and synaptophysin (Supplementary Figs S8 and S9) were also unchanged. A summary of the statistical analysis of western blot data is shown in Table 1. The loading controls (β-actin gels) are shown in Supplementary Fig. S10.

**Table 1:** Summary of western blotting statistical analyses.

Protein analyzed	ANOVA factor or Post-hoc test	Groups compared	F (DFn, DFd)	P value	Statistical significance?
PTEN	Interaction	Treatment × Genotype	F (1, 32) = 0.4084	0.5273	—
	Treatment	Control × IF	F (1, 32) = 0.7822	0.3831	—
	Genotype	WT × HT	F (1, 32) = 6.356	0.0169	Yes
	Holm-Sidak’s multiple comparisons test	C/WT × C/HT	—	0.1525	—
		IF/WT × IF/HT	—	0.5752	—
		C/WT × IF/WT	—	0.5877	—
		C/HT × IF/HT	—	0.8633	—
AKT	Interaction	Treatment × Genotype	F (1, 32) = 0.07327	0.7884	—
	Treatment	Control × IF	F (1, 32) = 0.1903	0.6656	—
	Genotype	WT × HT	F (1, 32) = 0.4513	0.5065	—
p-AKT T308/AKT	Interaction	Treatment × Genotype	F (1, 32) = 4.027	0.0533	—
	Treatment	Control × IF	F (1, 32) = 1.024	0.3191	—
	Genotype	WT × HT	F (1, 32) = 4.288	0.0465	Yes
	Holm-Sidak’s multiple comparisons test	C/WT × C/HT	—	0.0412	Yes
		IF/WT × IF/HT	—	0.9642	—
		C/WT × IF/WT	—	0.8422	—
		C/HT × IF/HT	—	0.1707	—
p-AKT S473/AKT	Interaction	Treatment × Genotype	F (1, 32) = 0.07327	0.7884	—
	Treatment	Control × IF	F (1, 32) = 0.1903	0.6656	—
	Genotype	WT × HT	F (1, 32) = 0.4513	0.5065	—
S6	Interaction	Treatment × Genotype	F (1, 32) = 0.02098	0.8857	—
	Treatment	Control × IF	F (1, 32) = 0.4574	0.5037	—
	Genotype	WT × HT	F (1, 32) = 1.428	0.2409	—
p-S6/S6	Interaction	Treatment × Genotype	F (1, 32) = 0.9075	0.3479	—
	Treatment	Control × IF	F (1, 32) = 0.7840	0.3825	—
	Genotype	WT × HT	F (1, 32) = 1.366	0.2512	—
AMPA	Interaction	Treatment × Genotype	F (1, 32) = 1.087	0.3050	—
	Treatment	Control × IF	F (1, 32) = 0.2580	0.6150	—
	Genotype	WT × HT	F (1, 32) = 0.7687	0.3872	—
NR1	Interaction	Treatment × Genotype	F (1, 32) = 0.8696	0.3580	—
	Treatment	Control × IF	F (1, 32) = 1.877	0.1803	—
	Genotype	WT × HT	F (1, 32) = 1.871	0.1809	—
NR2a	Interaction	Treatment × Genotype	F (1, 31) = 1. 367	0.2513	—
	Treatment	Control × IF	F (1, 31) = 0.03016	0.8633	—
	Genotype	WT × HT	F (1, 31) = 1.380	0.249	—
NR2b	Interaction	Treatment × Genotype	F (1,24) = 0.9843	0.3310	—
	Treatment	Control × IF	F (1,24) = 0.2853	0.5981	—
	Genotype	WT × HT	F (1,24) = 1.885	0.1825	—
PSD-95	Interaction	Treatment × Genotype	F (1, 32) = 1.896	0.1781	—
	Treatment	Control × IF	F (1, 32) = 0.7113	0.4053	—
	Genotype	WT × HT	F (1,32) = 1.054	0.3123	—
Synaptophysin	Interaction	Treatment × Genotype	F (1, 31) = 0.5522	0.4630	—
	Treatment	Control × IF	F (1, 31) = 1.313	0.2606	—
	Genotype	WT × HT	F (1, 31) = 0.08962	0.7667	—

## Discussion

We chose intermittent fasting as a tool to study the effects of *Pten* haploinsufficiency on the CNS as it is an environmental intervention that is known to influence many relevant pathways that modulate CNS structure and function and thus has the potential to stimulate mutant animals and possibly rescue the expected phenotypic deficits. In our study, IF did not influence the gain in body mass over time, even though the IF groups presented intermittent body mass loss on fasting days (Fig. 1a). Despite the significantly lower average food consumption (Fig. 1b), the absolute food consumption of the IF animals was biologically very similar to that of the control mice, considering that the IF animals were under conditions of complete fasting for half of the time. This similarity indicates that, on *ad libitum* food days, animals from the IF groups consumed almost twice as much food as control mice, an effect that could explain the counterbalance in the body mass after refeeding. Indeed, Anson *et al*.^31^ also observed that mice under IF conditions consumed similar amounts of food as control animals, thus preserving body mass^31^. However, food consumption and body mass control profiles are markedly variable in the literature, a variation that could have its origin in many factors, such as genetic background, housing conditions, energy source and palatability of the diet offered^32^.

Macrocephaly is remarkably prevalent in a subset of autism spectrum disorders^33^ that is frequently associated with *Pten* mutations^25^. Previous studies of *Pten* knockout models have shown that these animals present a macrocephalic phenotype^16,18,20,34^. We observed that HT mice were macrocephalic (Fig. 2a), particularly due to an increase in cortical mass (Fig. 2c). However, intermittent fasting did not alter the brain nor cortical mass, thus, not implicating IF in gross anatomical alterations.

Contrary to that previously reported by others^16,18,20,34^, we found no alterations in hippocampal or cerebellar mass in *Pten* neuronal haploinsufficient mice (Supplementary Fig. S2). This contrast could be derived from methodological particularities, since variabilities in PTEN studies in the literature have already been associated with differences in the genetic background of mouse models^35^. Kwon *et al*.^18^ and Backman *et al*.^16^, for instance, studied homozygous *Pten* deletion by using *Cre* expression directed by the *Gfap* (glial fibrillary acidic protein) promoter. Kwon *et al*.^20^ and Napoli *et al*.^34^, alternatively, used a model similar to that used in our study (*Nse-Cre*), assessing homozygous and heterozygous *Pten* deletion, respectively. In addition, the age of the animals used for these analyses also varied (from 2 through 29 weeks of age), adding another important variable.

Many groups observed an increase in locomotor activity of animals with neuronal *Pten* deletion^20,26,36,37^, although the animals might have avoided the central area of the open field^20,38^. In our study, even though we observed no differences in mobility (Fig. 4a,b,c), we found that neuronal *Pten* deletion decreased central area exploration when animals were under IF conditions, a result that could be individually interpreted as an anxiogenic effect. Interestingly, HT animals exhibited a greater exploratory profile in the elevated plus maze assay than WT animals (Fig. 3), a behavior that has also been observed by others^20,26^, although Smith *et al*.^36^ did not corroborate it, even in response to a stressful stimulus^36^. This effect, in contrast, is usually interpreted as an anxiolytic indicator. Therefore, these data from the elevated plus maze and open field assays, although supposedly paradoxical, seem to be corroborated by other studies.

The apparently controversial observed anxiety behavior assessed through these tests is not an uncommon event, and supposed inconsistencies have already been published and discussed previously^39,40^. This circumstance could have been due to the influence of the circadian rhythm or daily variations in the state of animal behavior at the moment of assessment, in addition to the effect of many particularities and factors of anxiety behavior that are differently observed by each behavioral assay^41^. Therefore, these disparities not only do not invalidate the results but actually highlight the importance of a further dissection of the anxiety-associated characteristics in this context, including the assessment of impulsivity and risk-taking behaviors.

Discrepant results have been reported regarding spatial memory in *Pten* knockout mice. A memory deficit in the Morris water maze was originally described by Kwon *et al*.^20^ in homozygous *Nse-Cre*-driven *Pten*-deleted animals^20^. However, in *GFAP-Cre Pten*-deleted HT mice, Smith *et al*.^36^ did not observe any learning or memory changes, even in kainate-challenged animals^36^. In our study, all animals effectively learned the platform’s position (Fig. 5a) and similarly retained the memory of the target quadrant for up to 4 consecutive days post-training, suggesting similar learning and extinction patterns between the different genotypes as well as the different feeding regimens (Supplementary Fig. S4).

Thus, with this water maze protocol, animals did not show significant spatial memory extinction, an indication that the assay parameters, *e*.*g*., number of training trials and time interval between probes, although satisfactorily induced learning of a spatial memory paradigm (*i*.*e*., platform localization in a water maze based on environmental spatial cues), might have been too strong to dissociate possible – if any – spatial memory effects induced by neuronal *Pten* haploinsufficiency. Additionally, we were unable to observe any effect of *Pten* conditional deletion on recognition memory (Supplementary Fig. S5), reinforcing the notion that specific parameters and/or conditions might be required to better elucidate the occurrence of cognitive deficits induced by a partial or total absence of neuronal PTEN. Other studies observed a deficit in social recognition memory^20,26,34^, indicating that these mice might indeed have an impairment in this memory type, although whether this effect is exclusive to social behavior or if it could be generalized to inanimate object recognition is unclear.

Similarly, there is also no consensus on the effect of PTEN deletion on fear memory. While mice with neuronal PTEN deletion directed by the GFAP promoter on a *status epilepticus* protocol showed increased learning in the fear conditioning test^36^, the original description of homozygous deletion through *Nse-Cre* mice by Kwon *et al*.^20^ did not show fear conditioning alterations^20^. In our study, however, HT mice exhibited an impairment in the passive avoidance test (Fig. 6), suggesting that strong aversive stimuli such as those that are fear-dependent are required to detect subtle behavioral anomalies induced by neuronal *Pten* haploinsufficiency. Although these behavioral tests – passive avoidance and fear conditioning – assess different types of fear memory, the complex circuitry that supports fear-associated processes appears to have a common mechanism of learned fear encoding^42^.

Interestingly, this fear-dependent memory impairment was effectively reversed by IF, emphasizing the modulatory potential of such environmental interventions in modulating the behavioral profile in this model. Notably, anxiety behavior is closely associated with learning and memory processes^43^. Therefore, we cannot rule out the possibility of an overlap between the anxiolytic/anxiogenic effect induced by neuronal PTEN haploinsufficiency under IF conditions and the observed fear memory outcome. Still, the basal latency to enter the dark zone was similar in all groups (Fig. 6), strengthening the hypothesis of a deficit in passive avoidance behavior in HT mice.

The western blots results confirmed reduced PTEN expression in the cortex (Fig. 7a) and increased AKT activation (p-AKT^T308^, Fig. 7b) in HT animals compared to those in WT animals. This effect was also confirmed in primary cortical neurons cultivated from WT and HT embryos (Supplementary Figs S11 and S12), which, together with the macrocephaly data (Fig. 2b), strengthen the validation of the model in our laboratory. However, we found no significant differences in p-AKT^S473^ or p-S6 levels, which are indicative of the activation state of the AKT signaling pathway. In addition, similarly to Smith *et al*.^36^, we found no differences in the levels of synaptic markers (PSD-95, synaptophysin) or glutamatergic receptors (AMPA, NR1, NR2a, NR2b), limiting the initial assessment of the molecular effects causing the observed behavioral phenotype. Considering that, in this model, the *Pten* heterozygous deletion is selective for mature neurons, normal PTEN levels and, consequently, unaffected downstream signaling in other cell types might mitigate the sensitivity of the western blotting assay. Therefore, future studies using this model should consider assessing molecular signaling effects through alternative approaches (*e*.*g*., immunofluorescence of brain tissues).

Borderline and paradoxical cognitive effects have frequently been observed in neuronal *Pten* haploinsufficiency and complete conditional knockout models. These borderline effects could stem from allostatic adaptations of affected signaling pathways and behaviors in these animals, highlighting that an external stimulation might be necessary to reveal defective phenotypes. Our findings support the modulatory role of neuronal PTEN in anxiety, learning and memory. We found that, in HT mice, IF uncovers both anxiolytic (elevated plus maze) and anxiogenic (open field) behaviors in parallel. Although no effect on spatial memory was observed, HT animals presented an impairment in fear memory – a phenotype interestingly rescued by IF, an environmental intervention, without differential effects on food consumption or brain mass. Considering our findings, we advise caution regarding the assumption of expected phenotypes of mutant animals. Further studies could benefit from environmental interventions or other stimuli to uncover hidden phenotype effects, thus allowing for a clearer and more comprehensive evaluation of the molecular mechanisms derived from *Pten* haploinsufficiency in neurons.

## Materials and Methods

### Animals and Intermittent Fasting

The *Pten*^*loxP/*+^; *Nse-Cre*^+^ lineage was originated by crossing *Pten*^*loxP/loxP*^ (donated by Dr. Antonio Di Cristofano from Albert Einstein College of Medicine, Bronx, NY, USA) and *Nse-Cre*^+^ mice (B6.Cg-Tg(Eno2-cre)39Jme/J, from Jackson Laboratory, Bay Harbor, ME, USA), within a C57Bl/6J background. Mice from the *Pten*^*loxP/*+^; *Nse-Cre*^+^ lineage were maintained in microisolator plastic cages in groups of up to 5 animals at 22 ± 2 °C in a 12-h light/dark cycle at the animal facility of the Laboratory of Molecular Neuropharmacology (Department of Pharmacology, Institute of Biomedical Sciences, University of São Paulo, São Paulo, Brazil). All experimental procedures were approved by and performed under the regulation of the Ethical Committee for Animal Research of the Institute of Biomedical Sciences (CEUA/ICB/USP, protocol #167, book 2, p. 167) and were in accordance with the guidelines of the *Sociedade Brasileira de Ciência em Animais de Laboratório* (SBCAL). In this study, animals of *Pten*^+*/*+^; *Nse-Cre*^+^, *Pten*^*loxP/*+^; *Nse-Cre*^*−*^, or *Pten*^*loxP/loxP*^; *Nse-Cre*^*−*^ genotypes were included in the wild-type (WT) group, as their *Pten* genes were not passive of Cre recombination. The neuronal PTEN heterozygous deletion (HT) group was consisted of *Pten*^*loxP/*+^; *Nse-Cre*^+^ mice (Supplementary Fig. S13). Considering the remarkable greater incidence of autism spectrum disorders in males^44^ and the sex-specific differences in stereotypical behavior of *Pten* haploinsufficient mice^45^, we chose to assess the effect of intermittent fasting (IF) and neuronal *Pten* haploinsufficiency on male mice.

The intermittent fasting (IF) protocol^46^ consisted of a daily alternation of *ad libitum* food followed by a day of complete fasting. Food was removed/replaced every day at 5:00 pm. The IF regimen was started when animals were 3–4 months old and was maintained for approximately 60 days, through the behavioral assays and up until euthanasia. Body mass and food consumption were assessed during this period. Animals were euthanatized by isoflurane overdose, and their brains were rapidly dissected, evaluated for wet mass (total brain, cortex, hippocampus, and cerebellum), and stored at −80 °C.

### Behavioral Tests

Behavioral analyses started 4 weeks after the beginning of IF and were conducted from the least to most stressful test (*i*.*e*., elevated plus maze, open field, novel object recognition, Morris water maze, and passive avoidance assays). All assays were recorded and analyzed with the ANY-maze Video Tracking Software (Stoelting Co., Wood Dale, IL, USA).

#### Elevated Plus Maze

Elevated plus maze was used to assess fear- and anxiety-associated behavior, based on Texel *et al*.^47^ with modifications. The apparatus consisted of a cross-shaped wooden maze (with 25 × 5-cm arms) elevated by a 60-cm support. Two opposite arms were surrounded by a 20-cm wall, while the other two were open (only with a 1-cm contention step). Mice were individually placed in the central area of the apparatus, facing one of the closed arms, and their mobility within the maze was assessed over 5 min. The exploration profile within the different areas of the maze (open arms, closed arms and center) was analyzed, and anxiety behavior was assessed by examination of the open arm exploration. Animals that fell from the apparatus had to be censored from the analyses. To avoid effects of acute fasting on fear and anxiety behaviors, the test was always conducted when IF animals were fed.

#### Open Field Test

The open field test was used to analyze fear- and anxiety-associated behavior, as well as exploratory behavior. The protocol was based on Kawamoto *et al*.^48^. Briefly, animals were allowed to freely explore for 10 min in a 40 × 40 × 15-cm plastic cage virtually divided into a central and peripheral area. Mobility was determined by distance travelled and mean speed. Anxiety-associated parameters were related to the central area exploratory profile. Cases when animals jumped out of the apparatus were censored from the analyses. Similar to the elevated plus maze, tests were conducted on days when IF animals were fed.

#### Morris Water Maze

The Morris water maze protocol was based on Shaw *et al*.^49^ and Okun *et al*.^50^. A circular pool was filled with water (27 ± 2 °C) rendered opaque by the addition of nontoxic white paint. A circular platform (9 cm of diameter) was submerged 1 cm below the water level in one of the pool quadrants. Environmental cues were placed in the surrounding room walls to facilitate spatial localization. Refractory animals – identified by stereotypical behaviors such as thigmotaxis and/or passive buoyancy – were censored from analyses. To avoid effects of acute fasting on learning and memory, the experimental design was planned to ensure that IF animals were fed on the longest test day (*i*.*e*., the last learning trial and 4-h probe day). Consequently, all other analyses had matched and balanced fast/fed days. *Learning period*: Animals were trained to find the platform location through four 60-s trials for 5 consecutive days. If an animal failed to locate the platform, it was gently directed toward it and allowed to rest on it for 10 s. Latency to find the platform, averaged by trial day, was analyzed and used as an indicator of spatial learning. *Spatial reference memory and memory extinction*: Animals were reintroduced to the water maze in absence of the platform 4 h, 24 h, 48 h, 72 h, and 96 h after the last training trial for 60 s. Persistent swimming within the area where the platform was placed during learning was used as a measure of spatial reference memory and extinction. *Spatial working memory*: The animals were prompt to learn and remember a different platform position over 4 testing days. The ability to match-to-place the new platform locations between the 4 intraday trials was used as an indicator of working memory. Latency to find the platform was averaged by trial across the different days.

### Passive Avoidance

The passive avoidance test was used to assess fear-associated learning and memory, based on the protocol from Vasconcelos *et al*.^51^. The apparatus consisted of a cage with two chambers, one dark and one bright, separated by an automated door and a stainless-steel grid floor with controlled electrification. During the exposure stage, mice were individually placed in the bright chamber and received an electric foot shock of 0.5 mA for 3 s after entering the dark chamber. After 24 h, mice were reintroduced to the bright chamber of the apparatus, and the latency to move to the dark chamber was used as a measure of fear-motivated short-term memory. Animals that were refractory to this test – i.e., mice that did not move to the dark chamber in the exposure stage – were censored from the analyses. The exposure stage was always conducted on a day when IF animals were fed so that learning of the shock context was not affected by acute fasting effects.

### Tissue Protein Extraction

Cortical cytosolic protein extraction was conducted using a protocol adapted from Vasconcelos *et al*.^51^. Briefly, tissues were homogenized in a glass-glass Dounce homogenizer in ice-cold lysis buffer (20 mM HEPES, 1.0 mM MgCl_2_, 0.5 mM EDTA, 1% NP-40, 1.0 mM EGTA, 0.5 mM PMSF, 2 g/mL leupeptin, 2 g/mL antipain, 3 mM Na_3_VO_4_, 20 mM sodium pyrophosphate) and centrifuged at 17,000 × g for 5 min at 4 °C. The supernatant was collected and stored at −80 °C for western blot analyses. Protein concentration was determined using the Bradford colorimetric method (#500-0006, Bio-Rad, Hercules, CA, USA)^52^.

### Western Blotting

Protein extracts were adjusted to a final concentration of 2.5 μg/μL in sample buffer (125 mM Tris-HCl, 4% SDS, 20% glycerol, 200 mM DTT, 0.02% bromophenol blue, pH 6.8) and subjected to SDS-PAGE electrophoresis as described by Laemmli^53^. Briefly, samples (25 μg) were separated using 10% polyacrylamide gels, transferred onto nitrocellulose membranes, blocked with 5% BSA, and incubated with primary antibodies against the following targets: PTEN (54 kDa, 1:1000, #9559 Cell Signaling Technology, Danvers, MA, USA), AKT (55 kDa, 1:2000, #sc-1619 Santa Cruz Biotechnology, Dallas, TX, USA), p-AKT^T308^ (60 kDa, 1:750, #4056 Cell Signaling), p-AKT^S473^ (60 kDa, 1:750, #550747 BD Biosciences, San Jose, CA, USA), S6 (32 kDa, 1:1000, #2217 Cell Signaling), p-S6 (32 kDa, 1:1000, #5364 Cell Signaling), AMPA (100 kDa, 1:500, #13185 Cell Signaling), NR1 (116 kDa, 1:500, #G8913 Sigma-Aldrich, Saint Louis, MO, USA), NR2a (180 kDa, 1:1000, #4205 Cell Signaling), NR2b (190 kDa, 1:1000, #4207 Cell Signaling), PSD-95 (95 kDa, 1:750, #sc-71933 Santa Cruz), synaptophysin (38 kDa, 1:2000, #4329 Cell Signaling), and β-Actin (42 kDa, 1:10000, #A5441 Sigma-Aldrich). Primary antibodies were diluted in 1% BSA, while secondary antibodies were diluted in 5% BSA (1:2000).

### Statistical Analyses

Results are expressed as the mean and standard deviation in graphs with bars or curves (parametric analyses) or as the median and quartiles in graphs with boxplots (non-parametric analyses). Except for curve data, the results are also represented as dot plots. Normality was assessed through the D’Agostino & Pearson omnibus normality test, and, for parametric analyses, outliers were detected and removed through the ROUT method (Q = 1%). Parametric analyses were conducted through single-measure or repeated-measures, when pertinent, two-way ANOVA followed by Holm-Sidak post hoc test. Non-parametric analyses were conducted through Kruskal-Wallis test followed by Dunn’s post hoc test. All statistical analyses were performed using GraphPad Prism version 6.01 for Windows (GraphPad Software, La Jolla, CA, USA). To facilitate comparison descriptions, groups were indicated as C/WT (control, WT), C/HT (control, HT), IF/WT (intermittent fasting, WT), or IF/HT (intermittent fasting, HT) in statistical results description and in the legends.

### Data Availability

All data generated or analyzed during this study are included in this published article and its Supplementary Information file.

## Electronic supplementary material

Supplementary material
