# Supplementary material for: Intermittent fasting uncovers and rescues cognitive phenotypes in PTEN neuronal haploinsufficient mice
Source: Sci Rep. 2018 Jun 5;8:8595. doi: 10.1038/s41598-018-26814-6 (PMC5988674; doi:10.1038/s41598-018-26814-6)
Supplement: Supplementary file 1 — Supplementary material [file 41598_2018_26814_MOESM1_ESM.pdf]

Intermittent fasting uncovers and rescues cognitive phenotypes in PTEN neuronal haploinsufficient mice

Cabral-Costa, J.V.; Andreotti, D.Z.; Mello, N. P.; Scavone, C.; Camandola, S.; Kawamoto, E.M.

SUPPLEMENTARY MATERIAL

SUPPLEMENTARY METHOD

Novel Object Recognition

This assay was based on Bevins and Besheer (2006)[1] and allowed for a cognitive assessment of learning and memory based on the conserved rodent behavior of predominantly exploring novel objects when paired with an already familiarized object. Animals were individually habituated to 40 x 40 x 15-cm plastic cages in 10-min sessions for 5 consecutive days. On the sixth day, two equal objects were attached to the cage, and animals were allowed to explore for 10 min. After a 2-h interval, mice were reintroduced to the cage, one of the objects was replaced with a new one of different shape and color, and exploration was monitored for 5 min. The time of head exploration of the area surrounding the objects was determined. The discrimination index was calculated as the ratio between the difference in exploration of the novel and the familiar objects over total time of object exploration. The experimental design was planned in a way that IF animals were fed on the probe day.

Pten$^{loxP/+}$*;Cre*$^{+}$ mice genotyping

After weaning, animals were marked with an ear clip and the tail tip (1-2 mm) was collected using a sharp scissor. For embryo genotyping, tails were collected right after dissection and processed to further identification of individual culture genotype. DNA was extracted in 75 µL 25 mM NaOH, 0.2 mM EDTA for 1 h, 98 ºC, on an orbital shaker (900 rpm). An equal volume of 40 mM Tris HCl, pH 5.5, was added and the solution, centrifuged for 3 min at 1,500 x g, and the supernatant was collected and stored at -80 ºC. Samples were genotyped through polymerase chain reaction (PCR) with the following primers: loxP, 5’- -3' and 5’- -3'; Cre, 5’-TGCTTCTGTCCGTTTGCCGGT -3’ and 5’-GTGAAACAGCATTGCTGTCAC -3; and Cre internal control (oIMR7338, oIMR7339), 5’-CTAGGCCACAGAATTGAAAGATCT-3’ and 5’-GTAGGTGGAAATTCTAGCATCATCC-3’.

Primary Embryonic Neuronal Culture (Cortex)

Primary neuronal cultures were prepared from cerebral cortices of embryonic day (E)16,5 from embryos of *Pten*$^{loxP/+}$*;Cre*$^{+}$ mice. Embryos were euthanized by decapitation with a sharp scissor and dissected. After meninges were removed, cortices were digested with 0.05% trypsin for 20 min at 37

°C. Cells were dissociated with a glass pipette at the presence of DNAse (Sigma-Aldrich, 9003-98-9). Dissociated cells were plated at a density of 1.0 x 10^5 cells/mL on Polyethylenimine (PEI, Sigma-Aldrich, 9002-98- 6)-coated wells and cultured in MEM (Vitrocell) containing 10% FBS for 3 h. After seeding, the medium was changed to Neurobasal medium (ThermoFisher, 21103049) supplemented with B-27 (ThermoFisher, 17504044), 0.5 mM glutamine and 1% Penicillin/Streptomycin. Cells were incubated at 37 °C in a humidified chamber at 95% air and 5% CO_2 , and the cultures were used for experiments after 14 days in vitro (DIV).

Immunofluorescence

The immunofluorescence protocol followed the method described previously[2]. In brief, cells were fixed in 4% formaldehyde for 15 min at 4°C before incubation in blocking solution (5 % donkey serum; 0.01 % Triton X-100 in PBS) for 1 h at 25°C. Cells were incubated with rabbit anti-PTEN antibody (1:200, Cell Signaling Technology, 138G6), rabbit anti-p-AKT^{T308} antibody (1:100, Cell Signaling Technology, 24469) and mouse Pan-neuronal Marker (1:1000, Millipore, MAB2300) overnight at 4°C. The cells were washed three times for 10 min with PBS and then incubated with anti-rabbit Alexa Fluor 488 (1:1000, Life Technology, A21206) and anti-mouse Alexa Fluor 594 (1:1000, Life Technology, A21203) for 2 h at 25°C. DAPI (1:25,000) was used to stain nuclei. Images were captured under a fluorescence microscope (Nikon 80i, Imaging Software - NIS Element V4.6) using a 20X objective and analyzed in the ImageJ (NIH) software.

Immunofluorescence Measurement

Immunofluorescence measurement was performed as described previously[3]. In brief, using ImageJ, a circle was drawn around each cell, as well around several adjacent background regions, and the parameters needed from each one were measured. The total corrected cellular fluorescence (TCCF) was determined as TCCF = integrated density – (area of selected cell x mean fluorescence of background readings). TCCF was calculated for approximately 40-60 cells from each embryo, which is represented by the average of all the obtained values. Only Pan-neuronal positive cells were analyzed. In the merged figures, only DAPI and Pan-neuronal signal were color-intensified in order to better clarify fluorescence; PTEN and p-AKT^{T308} fluorescence were presented without any modification.

SUPPLEMENTARY REFERENCE

1 Bevins, R. A. & Besheer, J. Object recognition in rats and mice: a one-trial non-matching-to-sample learning task to study "recognition memory". *Nat. Protoc.* **1,** 1306–1311 (2006).

2 Kawamoto, E. M.; Munhoz, C.D.; Lepsch, L.B.; de Sá Lima, L.; Glezer, I.; Markus, R.P.; de Silva, C.L.; Camarini, R.; Marcourakis, T.; Scavone, C. Age-related changes in cerebelar phosphatese-1 reduce Na,K-ATPase activity. *Neurbiol. Aging* **29**, 1712-20 (2007).

3 McCloy, R. A.; Rogers, S.; Caldon, C. E.; Lorca, T.; Castro, A.; Burgess, A. Partial inhibition of Cdk1 in G 2 phase overrides the SAC and decouples mitotic events. *Cell Cycle* **13**, 1400-12 (2014).

SUPPLEMENTARY FIGURES

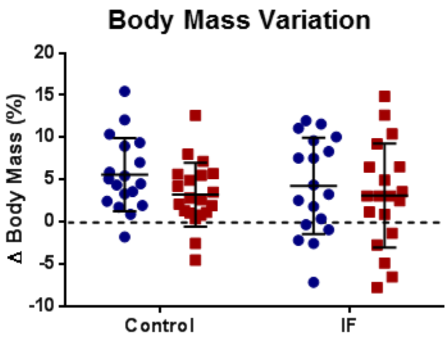

Supplementary Figure S1. Body mass variation after the intermittent fasting protocol. Two-way ANOVA followed by Holm-Sidak's post hoc test, P > 0.05, n = 18 (C/WT, IF/WT), 19 (IF/HT), or 20 (C/HT).

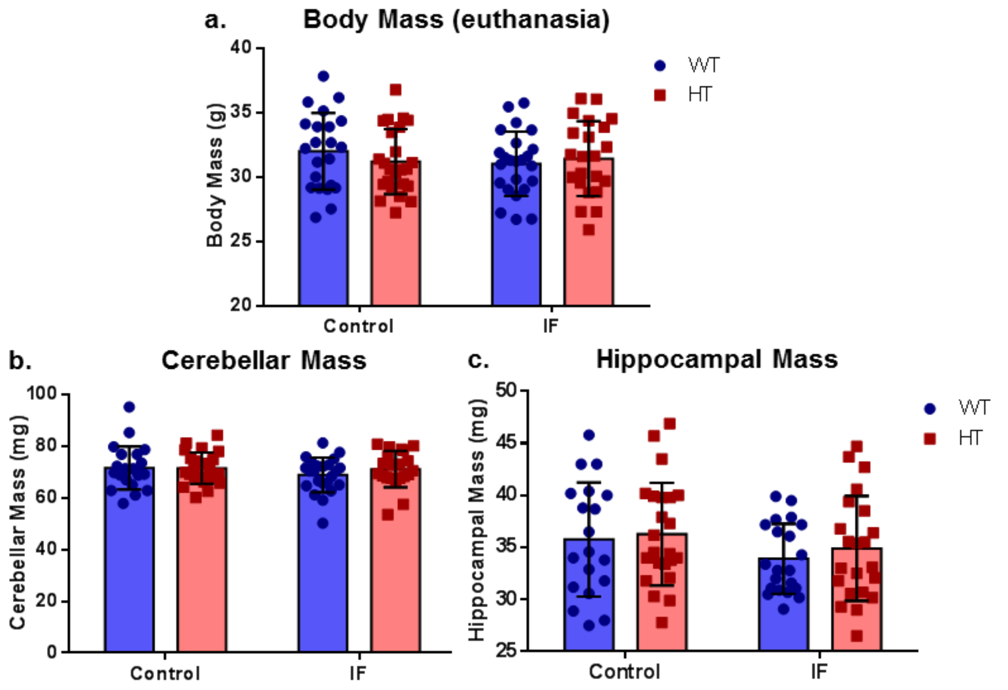

Supplementary Figure S2. Macrocephaly was not associated with body mass alterations or with cerebellar or hippocampal mass gain. (a) Body mass at the time of euthanasia, two-way ANOVA followed by Holm-Sidak's post hoc test, P > 0.05, n = 22 (C/WT, IF/HT) and 24 (C/HT, IF/WT); (b) cerebellar mass, two-way ANOVA followed by Holm-Sidak's post hoc test, P > 0.05, n = 22 (C/WT, IF/HT), 23 (IF/WT), and 24 (C/HT); (c) hippocampal mass, two-way ANOVA followed by Holm-Sidak's post hoc test, P > 0.05, n = 19 (C/WT), 21 (IF/WT), 22 (IF/HT), and 24 (C/HT).

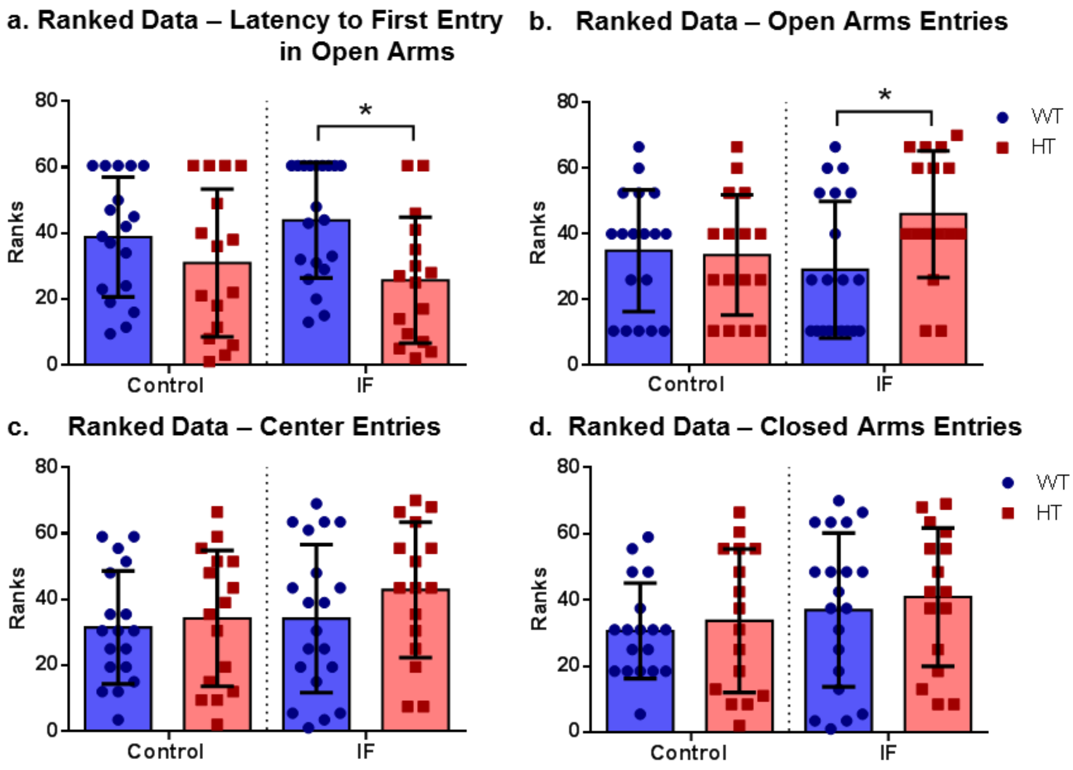

Supplementary Figure S3. Elevated plus maze data ranks. Ranked data from Kruskal-Wallis test for the (a) latency to first entry in open arms, (b) number of open arm entries, (c) number of central area entries, and (d) number of closed arm entries, * P ≤ 0.05, n = 16 (C/HT, IF/HT), 18 (C/WT), and 20 (IF/WT).

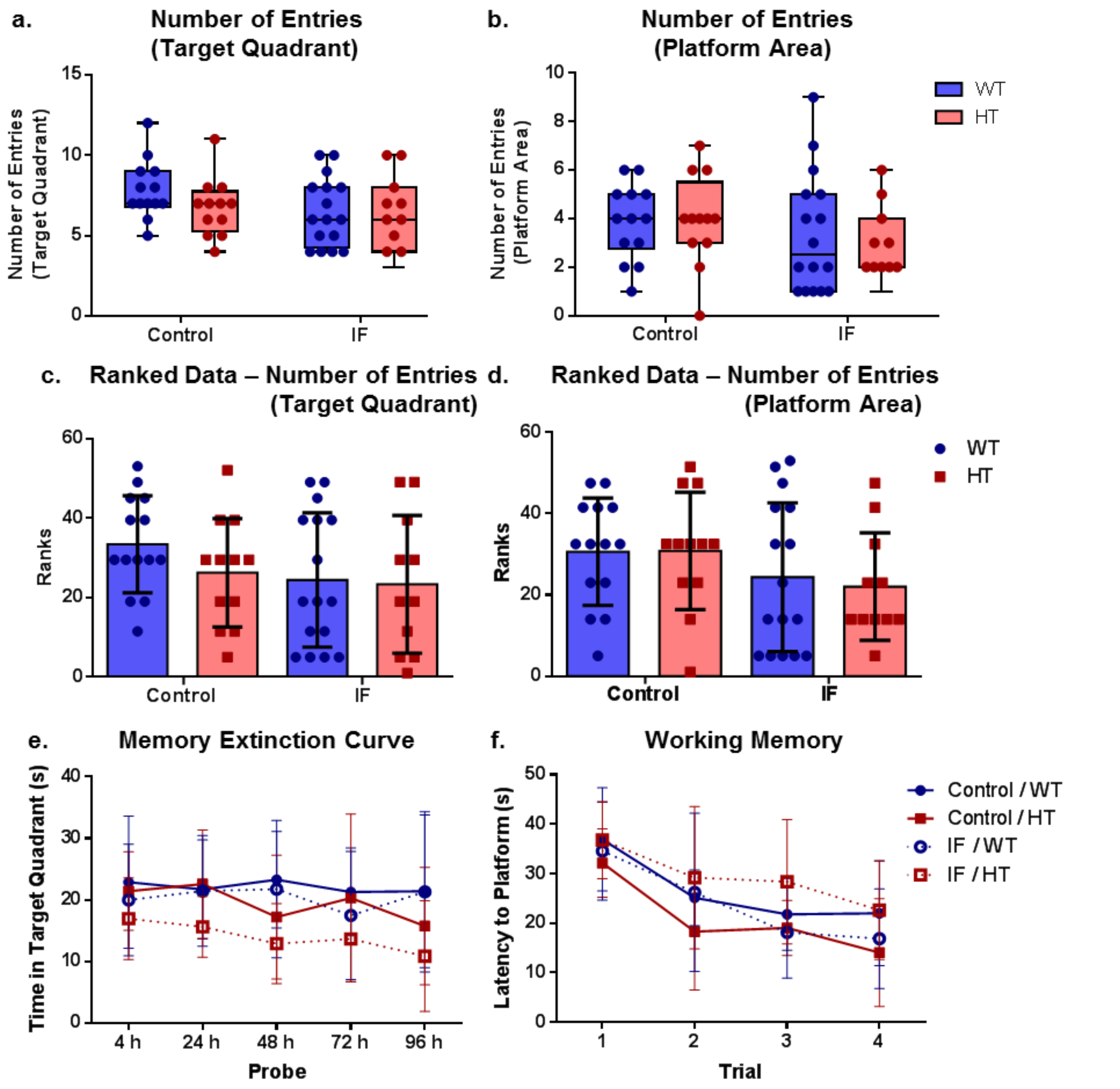

Supplementary Figure S4. Spatial memory, memory extinction, and working memory tests. (a) Number of entries in the target quadrant, Kruskal-Wallis test, $P > 0.05$, $n = 11$ (IF/HT), 12 (C/HT), 14 (C/WT) and 16 (IF/WT); (b) number of entries in the platform area, Kruskal-Wallis test, $P > 0.05$, $n = 11$ (IF/HT), 12 (C/HT), 14 (C/WT) and 16 (IF/WT); (c) ranked data from Kruskal-Wallis test for the number of entries in the target quadrant; (d) ranked data from Kruskal-Wallis test for the number of entries in the platform area, Kruskal-Wallis test; (e) memory extinction curve, two-way ANOVA with repeated measures followed by Holm-Sidak's post hoc test, $P > 0.05$, $n = 8$ (C/HT, IF/HT) and 9 (C/WT, IF/WT); (e) working memory learning curve, two-way ANOVA with repeated measures followed by Holm-Sidak's post hoc test, $P > 0.05$, $n = 7$ (C/HT, IF/HT), 8 (C/WT) and 11 (IF/WT).

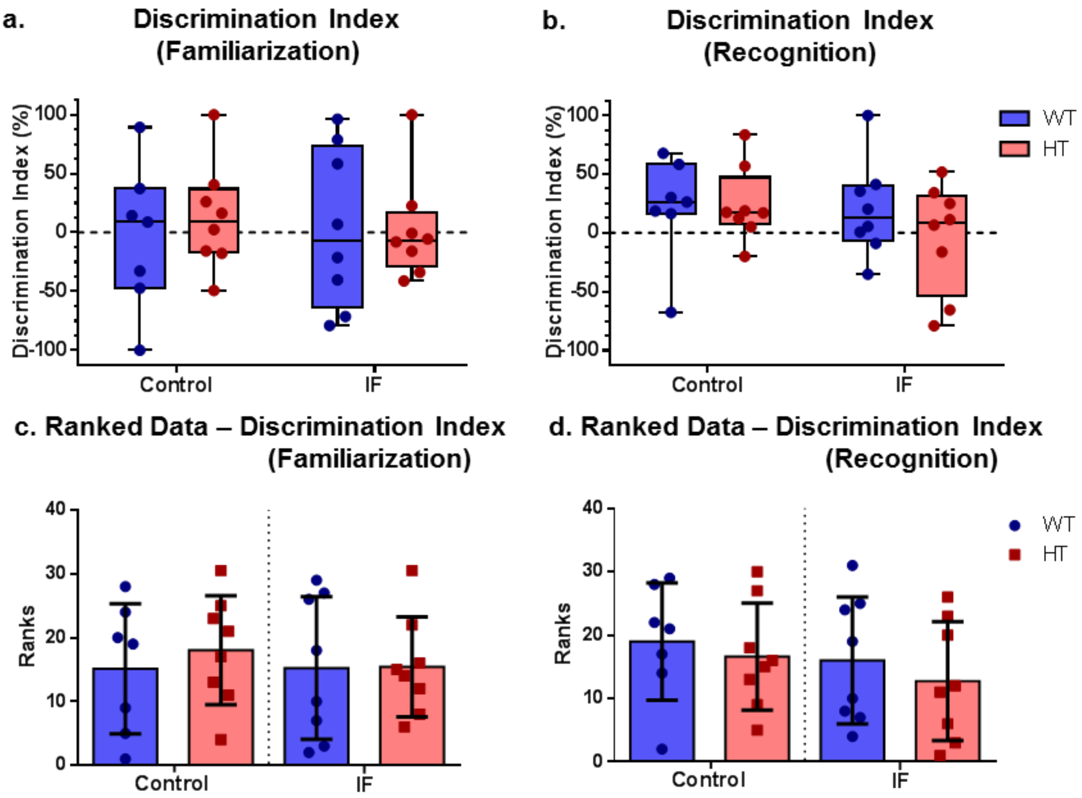

Supplementary Figure S5. Novel object recognition assay. (a) Discrimination index in the familiarization stage; (b) discrimination index in the recognition stage; (c) ranked data from Kruskal-Wallis test for the discrimination index in the familiarization stage; (d) ranked data from Kruskal-Wallis test for the discrimination index in the recognition stage, Kruskal-Wallis test, $P > 0.05$, n = 7 (C/WT) and 8 (C/HT, IF/WT, IF/HT).

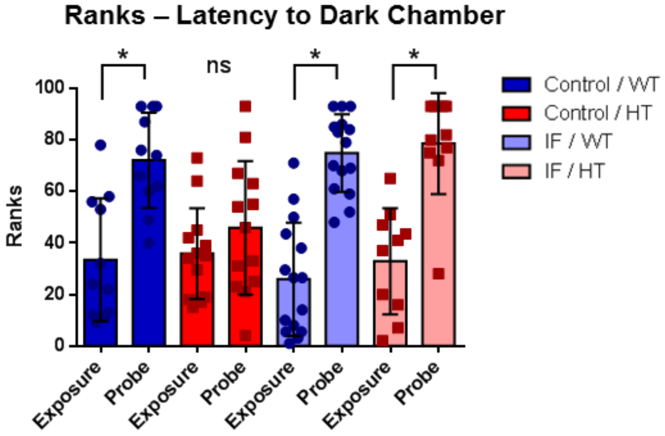

Supplementary Figure S6. Passive avoidance test data ranks. Ranked data from Kruskal-Wallis test for the passive avoidance test, * $P \leq 0.01$, n = 10 (IF/HT), 11 (C/WT), 13 (C/HT) and 15 (IF/WT).

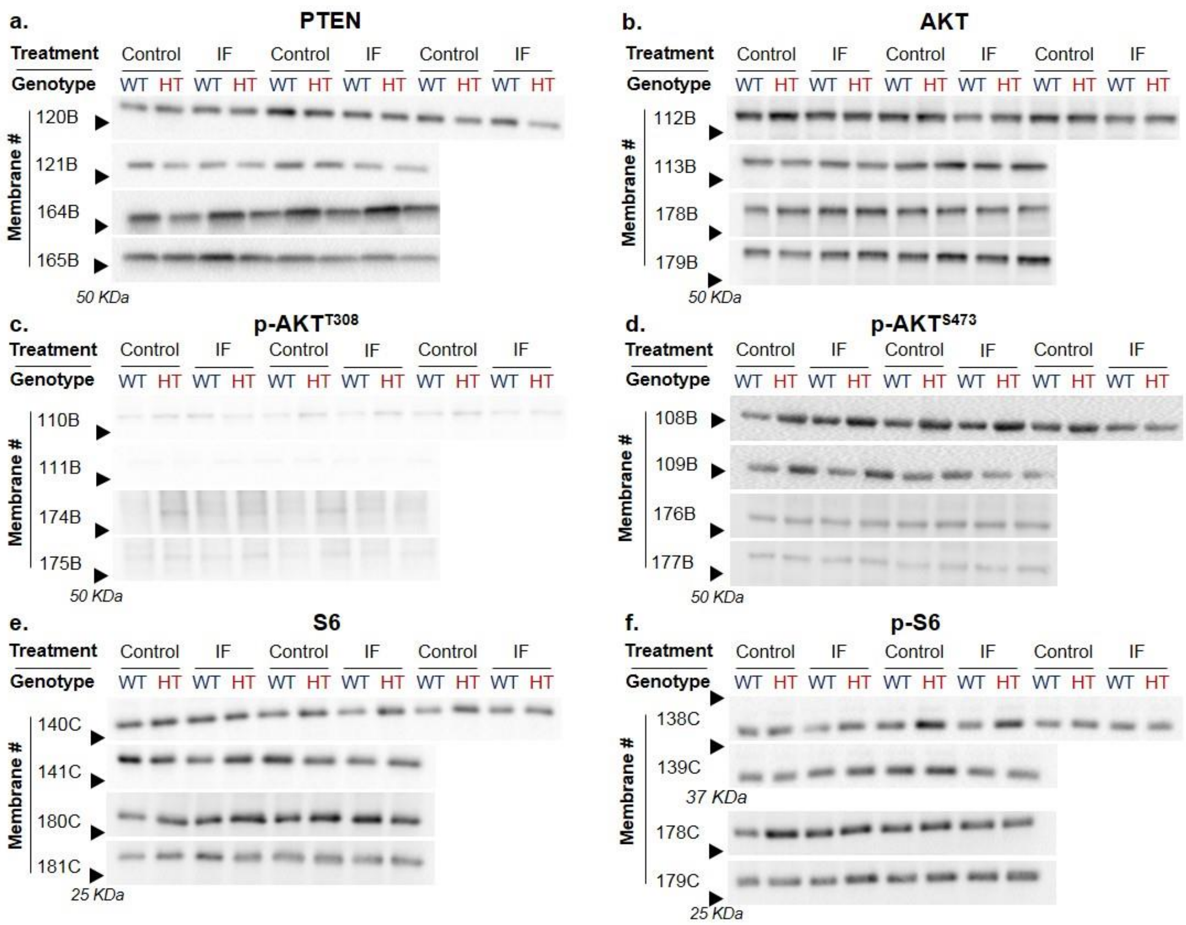

Supplementary Figure S7. Western blotting membrane images for the PTEN/AKT signaling pathway markers. (a) PTEN (54 kDa); (b) total AKT (55 kDa); (c) p-AKT^{T308} (60 kDa); (d) p-AKT^{S473} (60 kDa); (e) total S6 (32 kDa); (f) p-S6 (32 kDa). Membrane identification numbers are indicated on the left side of the panels. Approximate position of the molecular weight standard is indicated by a black arrowhead.

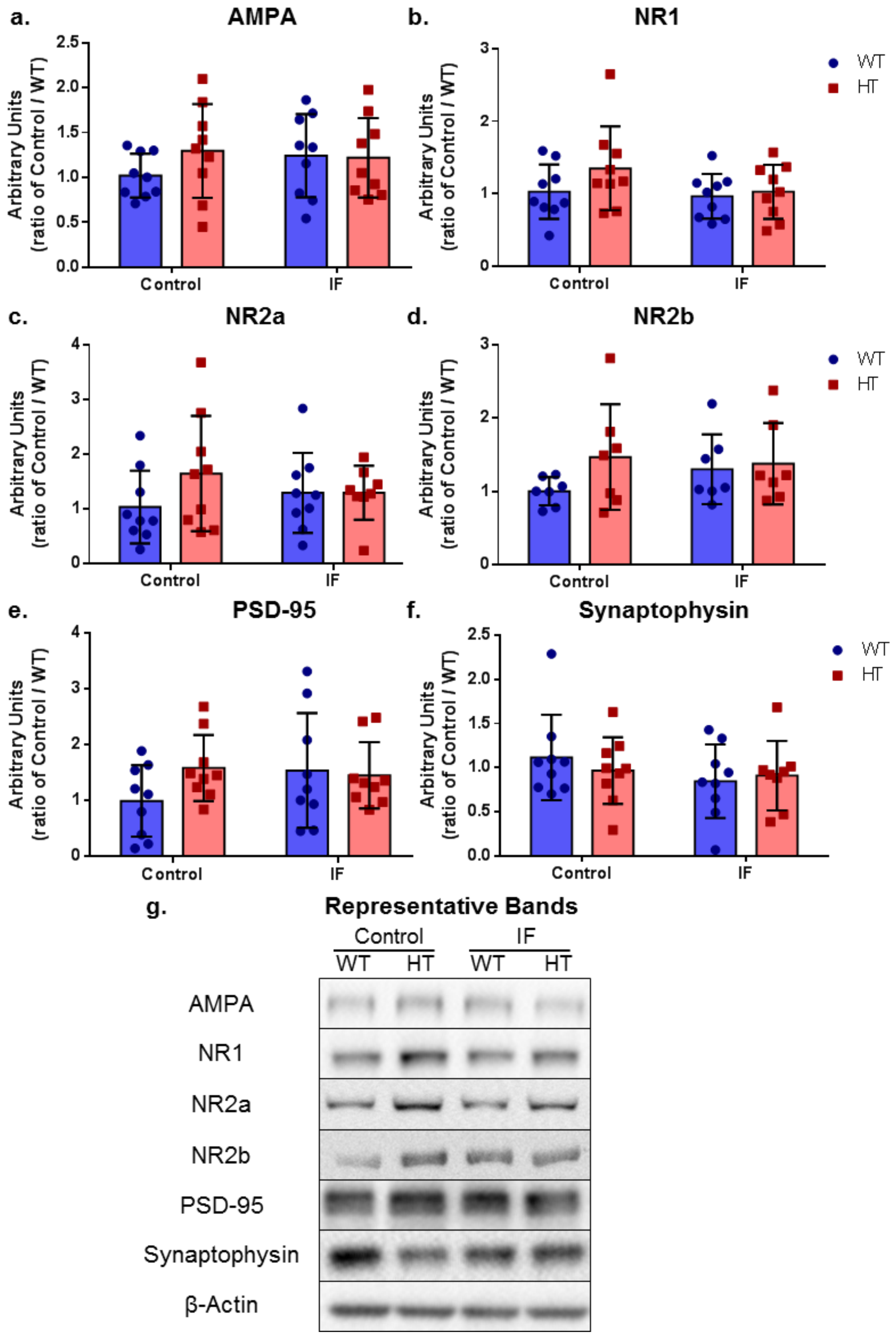

Supplementary Figure S8. Glutamatergic and synaptic markers. Protein levels assessed through western blotting. (a) AMPA receptor, n = 9 for all groups; (b) NMDA subunit NR1, n = 9 for all groups; (c) NMDA subunit NR2a, n = 8 (IF/HT) or 9 (C/WT, C/HT, IF/WT); (d) NMDA subunit NR2b, n = 7 for all groups; (d) PSD-95, n = 9 for all groups; (e) synaptophysin, n = 8 (IF/HT) or 9 (C/WT, C/HT, IF/WT); (f) representative bands. Two-way ANOVA followed by Holm-Sidak's post hoc test, P > 0.05. Full length blots are provided in the Supplementary Figures S9 and S10.

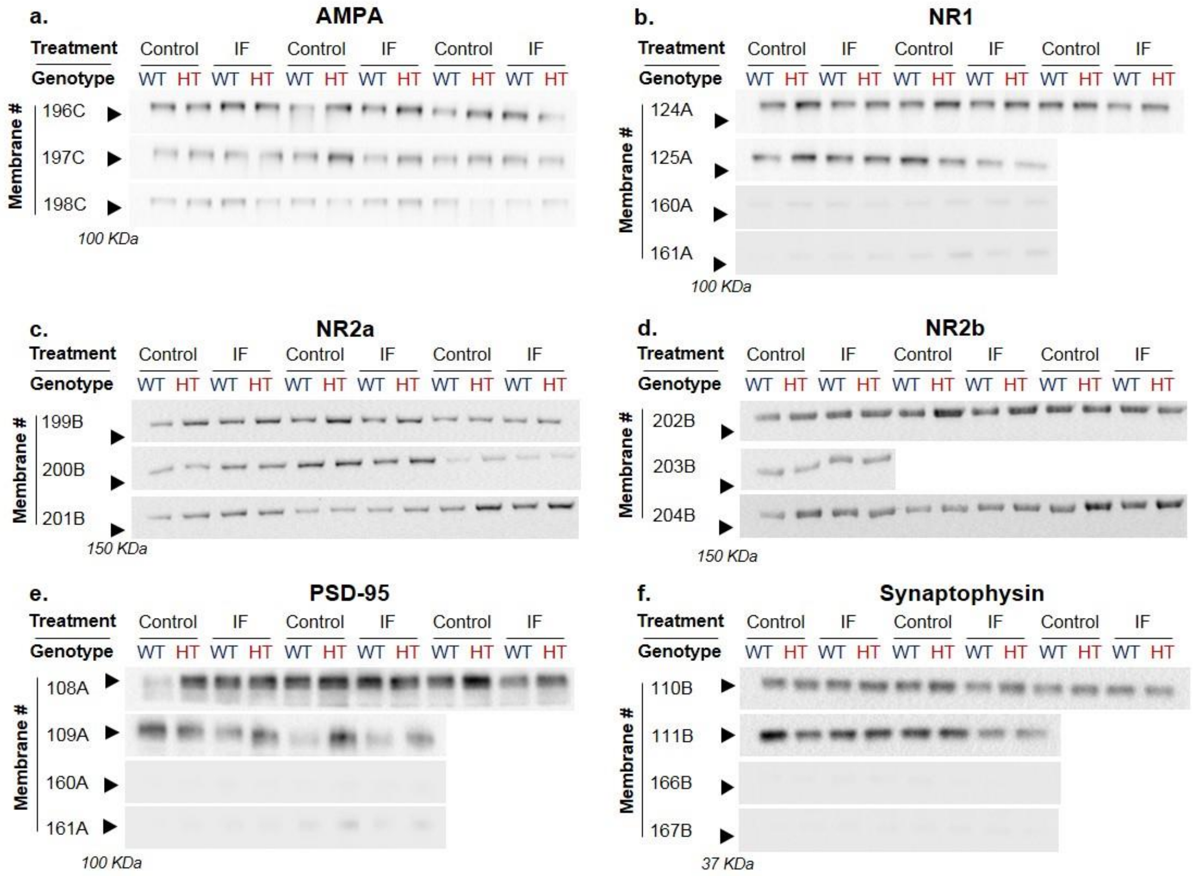

Supplementary Figure S9. Western blotting membrane images for the glutamatergic and synaptic markers. (a) AMPA receptor (100 kDa); (b) NMDA subunit NR1 (116 kDa); (c) NMDA subunit NR2a (180 kDa); (d) NMDA subunit NR2b (190 kDa); (d) PSD-95 (95 kDa); (e) synaptophysin (38 kDa). Membrane identification numbers are indicated on the left side of the panels. Approximate position of the molecular weight standard is indicated by a black arrowhead.

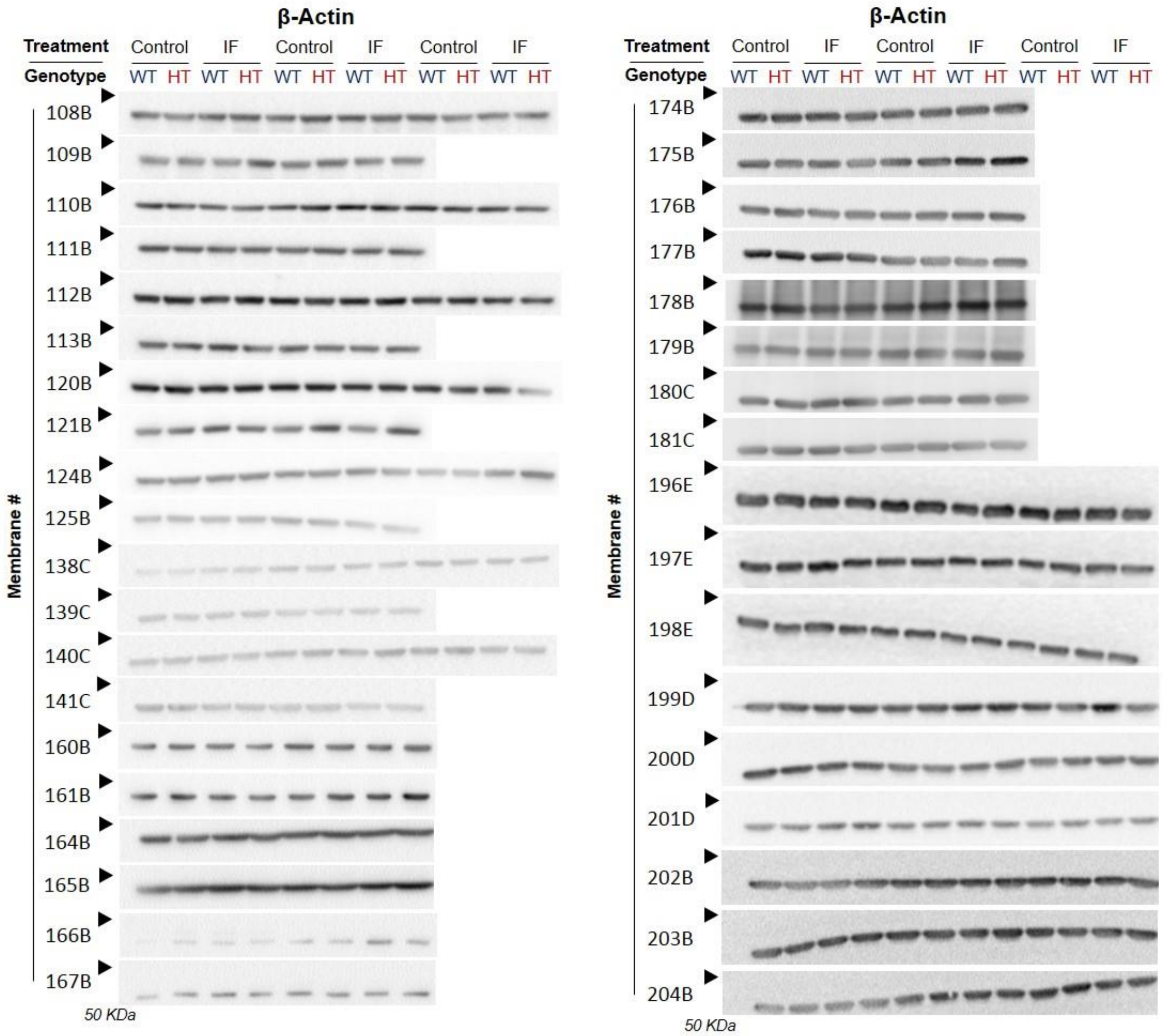

Supplementary Figure S10. Western blotting membrane images for the β-Actin marker (42 kDa). Membrane identification numbers are indicated on the left side of the panels. Approximate position of the indicated molecular weight standard is indicated by a black arrowhead.

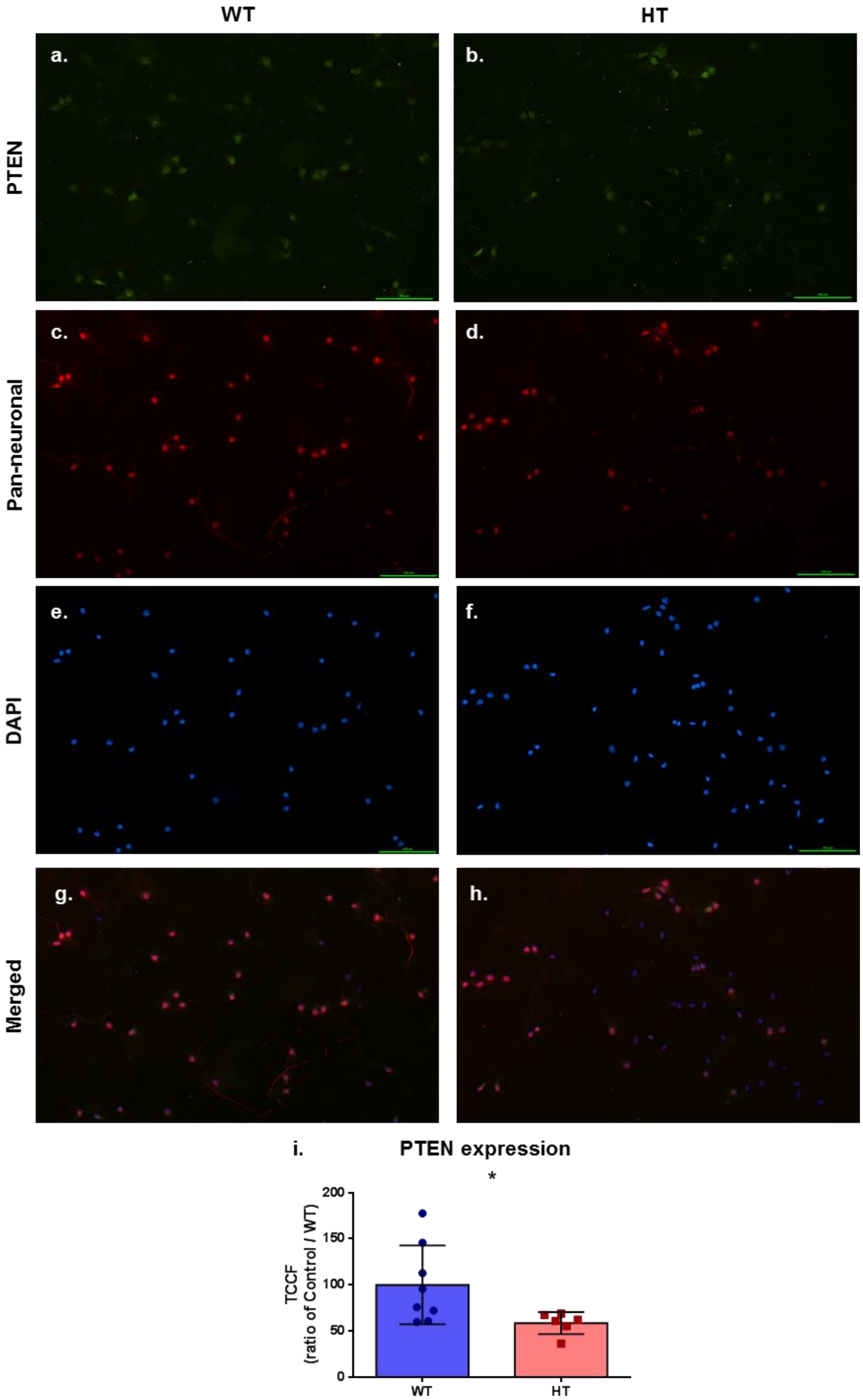
WT
HT
PTEN
Pan-neuronal
DAPI
Merged
a.
b.
c.
d.
e.
f.
g.
h.
i.
PTEN expression
*
TCCF
(ratio of Control / WT)
200
150
100
50
0
WT
HT

Supplementary Figure S11. Immunofluorescence for PTEN expression. (a-h) Representative images of PTEN (a, b), Pan-neuronal (c, d), and DAPI (e, f) staining, and of the merged channels (e, f), 20x magnification, scale bar: 100 μm; (i) quantification of the total corrected cellular fluorescence (TCCF) for PTEN of Pan-neuronal positive cells. Unpaired, two-sided Student's t-test, * $P \leq 0.05$. n = 6 (HT) and 8 (WT).

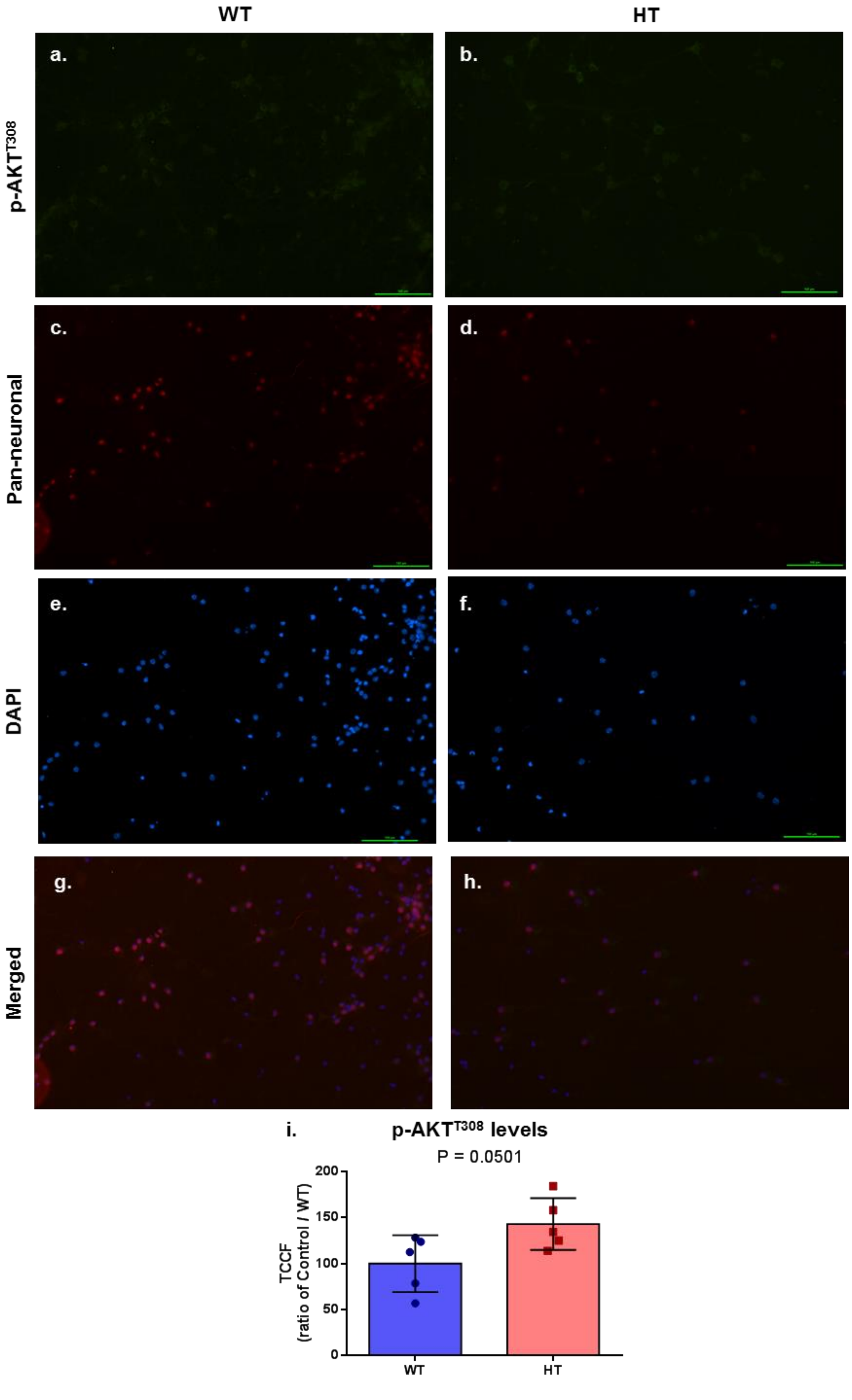
WT
HT
p-AKT T308
Pan-neuronal
DAPI
Merged
a.
b.
c.
d.
e.
f.
g.
h.
i.
p-AKT T308 levels
P = 0.0501
TCCF
(ratio of Control / WT)
200
150
100
50
0
WT
HT

Supplementary Figure S12. Immunofluorescence for p-AKT^{T308} levels. (a-h) Representative images of p-AKT^{T308} (a, b), Pan-neuronal (c, d), and DAPI (e, f) staining, and of the merged channels (e, f), 20x magnification, scale bar: 100 µm; (i) quantification of the total corrected cellular fluorescence (TCCF) for p-AKT^{T308} of Pan-neuronal positive cells. Unpaired, two-sided Student's t-test, P = 0.0501. n = 5 (WT, HT).

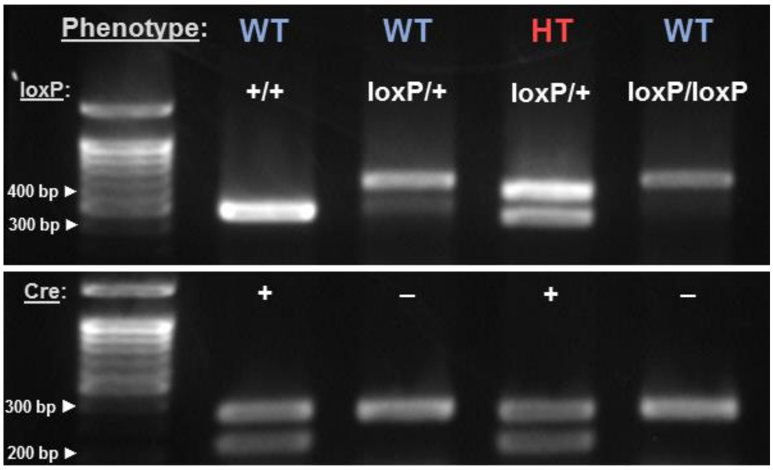

Supplementary Figure S13. Scheme of *Pten*$^{loxP/+}$*;Cre*$^{+}$ mice genotyping. The loxP floxed allele is perceived as a higher band in comparison to the WT allele. The Cre genotyping has a PCR control band (upper band) and a lower band that is present only in *Cre*$^{+}$ animals. Animals that does not express the Cre recombinase (Cre^{-}) were included in the WT group, as their *Pten* gene – even in *Pten*$^{loxP/loxP}$ or *Pten*$^{loxP/+}$ animals – is not recombined.
